# "Until death do us part". A multidisciplinary study on human- Animal co- burials from the Late Iron Age necropolis of Seminario Vescovile in Verona (Northern Italy, 3^rd^-1^st^ c. BCE)

**DOI:** 10.1371/journal.pone.0293434

**Published:** 2024-02-14

**Authors:** Zita Laffranchi, Stefania Zingale, Umberto Tecchiati, Alfonsina Amato, Valentina Coia, Alice Paladin, Luciano Salzani, Simon R. Thompson, Marzia Bersani, Irene Dori, Sönke Szidat, Sandra Lösch, Jessica Ryan-Despraz, Gabriele Arenz, Albert Zink, Marco Milella

**Affiliations:** 1 Department of Physical Anthropology, Institute of Forensic Medicine, University of Bern, Bern, Switzerland; 2 Institute for Mummy Studies, Eurac Research, Bolzano, Italy; 3 Dipartimento di Beni Culturali e Ambientali, PrEcLab—Laboratorio di Preistoria, Protostoria ed Ecologia Preistorica, Università degli Studi di Milano, Milano, Italy; 4 Ex-Soprintendenza per i Beni Archeologici del Veneto, Settore territorio, Sede di Padova-Nucleo di Verona, Padova, Italy; 5 Thompson Simon scavi e rilevamenti archeologici, Verona, Italy; 6 Soprintendenza Archeologia, Belle Arti e Paesaggio per le province di Verona Rovigo e Vicenza, Verona, Italy; 7 Dipartimento di Biologia, Università degli Studi di Firenze, Firenze, Italy; 8 Department of Chemistry, Biochemistry and Pharmaceutical Sciences and Oeschger Centre for Climate Change Research, University of Bern, Bern, Switzerland; University of the Witwatersrand, SOUTH AFRICA

## Abstract

Animal remains are a common find in prehistoric and protohistoric funerary contexts. While taphonomic and osteological data provide insights about the proximate (depositional) factors responsible for these findings, the ultimate cultural causes leading to this observed mortuary behavior are obscured by the opacity of the archaeological record and the lack of written sources. Here, we apply an interdisciplinary suite of analytical approaches (zooarchaeological, anthropological, archaeological, paleogenetic, and isotopic) to explore the funerary deposition of animal remains and the nature of joint human-animal burials at *Seminario Vescovile* (*Verona*, Northern Italy 3^rd^-1^st^ c. BCE). This context, culturally attributed to the Cenomane culture, features 161 inhumations, of which only 16 included animal remains in the form of full skeletons, isolated skeletal parts, or food offerings. Of these, four are of particular interest as they contain either horses (*Equus caballus*) or dogs (*Canis lupus familiaris*)–animals that did not play a dietary role. Analyses show no demographic, dietary, funerary similarities, or genetic relatedness between individuals buried with animals. Isotopic data from two analyzed dogs suggest differing management strategies for these animals, possibly linked to economic and/or ritual factors. Overall, our results point to the unsuitability of simple, straightforward explanations for the observed funerary variability. At the same time, they connect the evidence from Seminario Vescovile with documented Transalpine cultural traditions possibly influenced by local and Roman customs.

## Introduction

The deposition of whole animals or animal parts is an important component of funerary rituals among different human societies and frequently attested archaeologically by faunal remains found in burial contexts (e.g., [1–3] among others). The deposition of animal parts from taxa normally exploited for alimentary purposes, such as *suidae*, *caprinae*, and *bovinae* for Eurasian contexts, may point to their ritual offering as food to the deceased, a custom widely distributed geographically and chronologically [4]. However, the same interpretation is less satisfying in other instances, like the presence in burials of taxa usually absent from the menu of a given population, depositions of whole animals, or of animals unaccompanied by human interments. In all of these cases, the discussion of the archaeological evidence needs to take into account additional elements, including: (a) the symbolism associated with specific species in a given culture, (b) the possible selection of some animals as sacrifices, and (c) their social role and link with the deceased with whom they are buried. A typical find outside the notion of funerary food offering is the presence in burials of horses and dogs. Starting from its spread around 2200–2000 BCE from the Volga-Don region [5], the domestic horse (*Equus caballus*) quickly became economically and militarily central across Eurasian societies. One can link the appearance of horses in funerary and ritual contexts to their fast-growing importance and their role as a status symbol starting from the Eurasian Bronze Age (e.g., [6–11]).

Turning to dogs (*Canis lupus familiaris*), their appearance in human burials is archaeologically documented from at least the late Paleolithic, the earliest evidence of this custom being that of Born-Oberkassel (Germany, 12290–12050 cal. BC) [12, 13]; also see [14]). The appearance of dogs in funerary contexts, associated with human graves or not, presents a remarkable geographic distribution, with finds identified in Eurasia and the Americas and a chronological extension spanning from the Paleolithic to contemporary times [14–17].

Throughout the years, the interest in the cultural significance of horse and dog depositions has led to a number of contextual analyses and review papers (e.g., [6, 18, 19]). One survey by Prummel [19] on early medieval dog and horse burials in continental Europe and Anglo-Saxon England stands out due to its extensive documentation and analytical depth. Cross [18] also discusses the multiple meanings of horse burials in Britain during the first millennium CE, linking their presence in funerary spaces to the high status of the deceased, sacrificial rites, and, in some cases, feasting including the consumption of horse flesh. A similar association between the deceased’s social status and the presence of horse remains has also been suggested for burials in Lithuania dated between the 2^nd^-7^th^ centuries AD [6].

The works of Munt and Meiklejohn [17], Perri [20], and Morey and Jeger [14] provide methodological and interpretive reflections on the symbolic importance of dogs, dog burials, and joint dog-human interments. They clearly demonstrate the challenges in interpreting this evidence due to the cultural contingency and multiple facets (e.g., symbolic, religious, economic, and affective) of animal-human relationships.

Due to the lack of direct information, the opacity of the archaeological record is especially problematic for prehistoric contexts. Among these, the "Celtic" cultures distributed in Continental Europe and Britain during the Late Iron Age (⁓5^th^-1^st^ c. BCE) offer various archaeological examples of such complex animal-human relationships.

For the La Tène and early Romanization periods (ca. 4^th^-1^st^ c. BCE), the funerary and more general ritual role of animals has been discussed in different studies on enigmatic "Celtic" sites, mostly from France (e.g., Acy-Romance and Ribemont [21, 22]) and Switzerland (e.g., La Tène and Le Mormont [23, 24]). Conversely, little is known about the La Tène cultural groups of the Italian peninsula, with only a few examples of horses in burials [11], and there is little information on the economic relevance of the represented animal taxa (e.g., [25]).

Vitali [11] provides a review of these known horse depositions in the Italian peninsula during the Iron Age, which includes spectacular cases from Early Iron Age (Veneti) contexts (e.g., cf. [26–28], also see tomb 61 from Colombara di Gazzo Veronese [29]) as well as later evidence attributable to the Senones, another "Celtic" Transalpine group of Central Italy. Among the Veneti sites, the most famous case emerged from the necropolis of Piovego (6^th^-4^th^ c. BCE), where Burial 12 contained a young adult male and a horse. Both the human and the horse were oriented east-west and facing east, with the horse laying with its legs crossed under its chest and the neck bent while the man was laid on top in a supine position, possibly on a wooden litter [28, 30]. The sacrificing of horses and their burial, either in isolation or along with a deceased human, has also been discovered at other Venetic sites and interpreted as a ritual performed upon the death of high status individuals. Such examples include the isolated horse burials of Le Brustolade Altino (Venezia, Italy) [27, 31], Oderzo (Treviso) [32, 33], Este (Padova), Oppeano Veronese (Verona) [11] and the human-horse co-burials of via Tiepolo/S.Massimo (Padova) [34] and Este (Padova) [35]. An example from the Senones is the necropolis of Montefortino d’Arcevia (Ancona, Central Italy) where the presence of horse depositions is related to sacrificial practices [36]. Interestingly, this interpretation is indirectly supported by Caesar, who, in the *De Bello Gallico*, describes the Gallic custom of sacrificing the deceased’s animals alongside him or her on the funerary pyre (Caesar, *De Bello Gallico*, VI, 2, 19).

Compared with the Senones, the funerary appearance of horses among the Cenomani has so far been extremely limited, with only a single case from Carzaghetto di Canneto sull’Oglio (Mantova) [37]. However, this situation recently changed thanks to the discovery of human-horse and human-dog co-burials at the Late Iron Age (Cenomane) site of Seminario Vescovile (Verona, Northern Italy, 3^rd^-1^st^ c. BCE, henceforth "SV"). These new findings, presented and discussed in this work, are important for different reasons. First, they expand our still fragmentary knowledge about the funerary and cultural variability of this group. Second, the availability of detailed archaeological documentation together with an extensive anthropological and isotopic dataset [38–43] and preliminary human ancient DNA (aDNA) data [44] provide the basis for a solid contextualization of the funerary zooarchaeological evidence.

### Archaeological context

**Fig 1:**
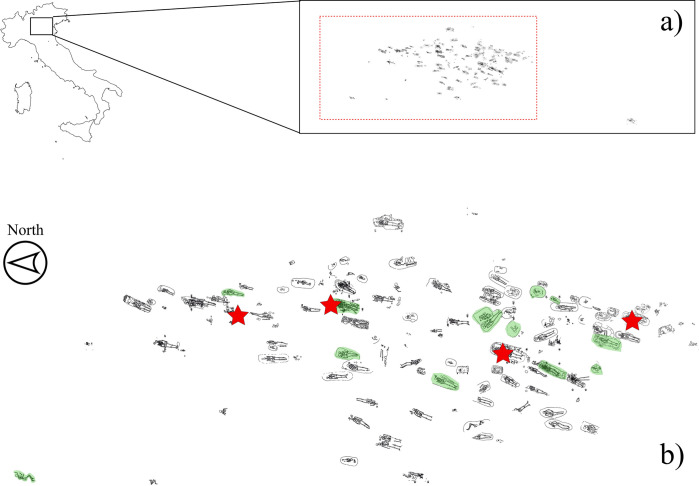
(a) geographical location of SV and overall view of the necropolis; (b) close-up showing the animal-human co-burials (red stars) and burials with food offerings (green shades) (map of Italy modified from https://www.fla-shop.com/svg/italy/ under a CC BY license. Plan by S.R. Thompson and M. Bersani, courtesy of SABAP-VR Soprintendenza archeologia, belle arti e paesaggio per le province di Verona, Rovigo e Vicenza).

The archaeological excavations in the courtyards of the Bishop’s Seminary of Verona took place between 2005 and 2009 in an area of over 2000 m^2^ on the eastern outskirts of the Roman town between a consular road (the *Via Postumia)* and a minor branch of the river Adige (Fig 1A and 1B) [45–48].

Between the 1^st^ and 3^rd^ century CE, the investigated area was an important center for metallurgical production and occupied by a series of buildings to the west of a Roman road. The site was also close to a sanctuary; numerous *favissae* (places of offerings) and votive deposits were uncovered during the excavation [49]. Beneath these structures was a necropolis of the local pre-Roman population (Cenomani), whose settlement was on the slopes of the hill of St. Peter on the left bank of the river Adige [38, 47]. The burial site included over 160 inhumations with grave goods dating to the late La Tène period [46, 48], and preliminary radiocarbon dating pointing to the 3^rd^ -1^st^ century BCE also confirmed this chronology [38, 39, 42]. In general, burials were simple pits, occasionally equipped with "funerary structures" composed of stones outlining the edge of the pit and/or covering the burial (cf. [39] for further details). Although none of the graves contained weapons, the funerary items were quite variable (e.g., pottery, pins, coins, rings, and a few knives), with some plates and small globular vessels exhibiting inscriptions in the Lepontic alphabet [50, 51]. Individuals at SV were mostly oriented north-south in a supine and extended position—rarely was the skeleton prone or on its side—and only 16 of these burials included faunal remains, either as fully articulated skeletons or as isolated parts.

A series of bioarchaeological studies has offered new insights about the lifestyle, social differentiation, and dietary patterns at SV [39, 42, 43]. The results of these works highlight differences between sexes in the performance of daily activities and overall exposure to biomechanical load [40] as well as a weak social differentiation and a homogenous exposure to developmental stressors during growth [39]. Stable isotope data of carbon and nitrogen pointed to an extended breastfeeding period [43], an almost exclusive consumption of C_4_ plants (possibly broomcorn and foxtail millet), and a higher proportion of animal proteins in the diets of males [42]. More recently, a preliminary study carried out on a subset of individuals has provided the first estimates of residential mobility at the site. Based on oxygen and carbon isotopic ratios, this work pointed to a low frequency of nonlocal individuals and to the Alpine area as a potential source of newcomers [42]. Compared with this extensive body of research on the human remains, no data have been available so far on the zooarchaeological finds associated with the SV burials.

### Research questions and methodological approach

From a funerary perspective, the preliminary evidence from SV raises the question of why, among such a large number of identified burials, only 16 (9.9%) revealed animal remains. Meniel [25] suggested a link between funerary heterogeneity and social differentiation in Gaul (modern-day France). We may therefore wonder if it is possible to detect any additional patterns (e.g. regarding diet, age at death, sex distribution, and genetic relatedness) linking the individuals buried with animals at SV. To address this question, we apply a multidisciplinary approach including zooarchaeology, paleogenomics, and geochemistry (stable isotopes and radiocarbon dating). Specifically:

1. Through estimations of sex, age at death, and other biometric and taphonomic characteristics, the zooarchaeological analyses aim to explore the taxonomic and demographic variability of the represented animals in order to provide insights about the dynamics leading to their deposition and their likely symbolic meaning (e.g., joint human-animal burials, food offering, etc.).
2. Paleogenomic analyses investigate possible genetic relatedness (kinship) among the individuals buried with animals.
3. The re-analysis of published human and animal stable isotope data from SV [39, 42] explores dietary (and possibly socioeconomic) differences between the individuals provided with animals *vs*. the rest of the buried population.

## Material and methods

All necessary permits were obtained for the described study, which complied with all relevant regulations (D.Lgs. 42/2004, art.21 of Ministero per i Beni e le Attività Culturali e per il Turismo- Soprintendenza Archeologia, Belle Arti e Paesaggio per le province di Verona, Rovigo e Vicenza (SABAP, Italy), permission number 6052 of 10.03.2020.

### Zooarchaeology and anthropology

The analyzed faunal and human archaeological remains are currently stored at the facilities of the Archaeological Superintendency in the city of Verona (Soprintendenza Archeologia, Belle Arti e Paesaggio per le province di Verona Rovigo e Vicenza, SABAP Verona, Italy).

Burials from SV featuring the presence of animal remains can be classified into two main groups: "Human-animal co-burial" and "Food offering". The distinction between these two categories is based on specific criteria. Human-animal co-burials include cases where humans are buried with animals that are mostly whole with preserved anatomical connections. This type of burial suggests that the animal held a status other than that of "food offering", which could moreover imply some degree of emotivity in the human-animal relationship [52]. This type of find stands out as a specific class of deposition because the act of burying a complete animal seems to symbolize the attribution of a social function/role in the society [53]. The animals associated with these symbolic contexts are primarily the horse and the dog, as already highlighted by Behrens [54] as the animals most frequently buried with human individuals. Their "social personality" is also highlighted by the fact that during the Iron Age and specifically in northeastern Italy, horses and dogs are also frequently buried alone; this is possibly linked to ceremonial reasons involving their sacrifice (see [28, 30, 33]). This interpretation could also pertain to the deposition of body parts, in particular the head, with probable symbolic value as *pars pro toto* (body part representing the whole) [55, 56]. Conversely, food offerings tend to be partial animal remains, possibly even in anatomical connection, that are typically exploited by humans as food resources. The main component of this second group is the pig (see below for further discussion).

The analyzed faunal remains show variable states of fragmentation and completeness. Whenever possible, fragments from the same bones were reattached after their cleaning in order to allow for their measurement. For taxonomic identification, we refer to Schmidt [57]. We estimated individual age at deaths based on Silver [58] and Levine [59] and osteometric measurements follow Von den Driesch [60]. Sex estimations followed the methods listed in Ruscillo [61]. For horses, we based our estimations on the presence or absence of the canine and the morphology of the pubic bone, while for dogs we relied on the presence of the *baculum* (os penis) [62]. We calculated horse and dog withers height following May [63] and Harcourt [64], respectively.

We presented the biological profiles for the humans from SV in previous works [38–40]. In these, we estimated adult age at death based on the morphological changes of the pubic symphysis and the auricular surface of the ilium, and if these skeletal elements were not available, using other methods (e.g. modification of the sternal end of the 4^th^ left rib and degree of wear of the dental crown) [65–69]. We estimated the age at death of non-adults based on the eruption of deciduous and permanent teeth, diaphyseal measurements, and the degrees of epiphyseal fusion [70–75]. Sex estimations in adults were determined based on the morphology of the pubic symphysis, coxal bones, and cranial and mandibular dimorphic traits following standard anthropological methods collected in Buikstra and Ubelaker [76].

### Human ancient DNA analysis

We collected bone powder from the inner part of the Pars Petrosa (PP) [77] of the human individuals buried with animals (n = 16). This step was carried out in a dedicated pre-PCR area of the ancient DNA (aDNA) laboratory of Eurac Research in Bolzano (Italy). Double-stranded genomic libraries were constructed [78] and sent to an external company (Macrogen Sequencing Centre, Seoul) for shotgun sequencing. Through bioinformatic analyses of sequenced reads, we assessed the authenticity and preservation of the aDNA of the samples. Even though all samples comply with the quality criteria for the performance of the enrichment reaction (human endogenous content > 1%), we randomly selected 11 samples for the enrichment of more than 1.3 million SNPs on the human genome [79, 80] and subjected them to sequencing and additional bioinformatic analyses to assess the authenticity of aDNA reads [81] (see S1 Text for more details). Contamination estimates were then performed on mtDNA and X-chromosome data [82, 83] whose thresholds were set to 5% and 3% respectively. The genetic sex was determined using shotgun data only and merged data (shotgun + capture) (see S3 Table). We used two different methods [84, 85] following the required minimum number of human reads of 1,000 and 100,000, to obtain a reliable estimation for [84, 85], respectively. Moreover, we inferred biological relatedness among individuals using three different methods: TKGWV2, READ, and KIN [86–88] (see S1 Text for details). For all methods, we followed the thresholds suggested by the authors: for the READ method, a threshold of 0.1X mean coverage of the reads mapping to the human reference genome [87], for the TKGWV2 method, the threshold of 0.026X average coverage along with 18,000 SNPs [86], and for the KIN method, the threshold of 0.5 X sequence coverage [88].

### Radiocarbon and stable isotope analysis

We analyzed the bone samples for ^14^C dating at the Laboratory for the Analysis of Radiocarbon with AMS (LARA) of the University of Bern, following slightly modified criteria from Szidat et al. [89], samples were prepared by implementing an ultrafiltration step, as recently performed in Steuri et al. [90] (see details in S1 Text). Radiocarbon ages were translated into calendar ages with OxCal 4.4 [91] using the IntCal20 calibration curve [92].

The isotopic (δ^13^C, δ^15^N) data of the human and some of the animal remains have been published elsewhere [42].

In this work, we repeated the isotopic analyses of four humans (US 2731, US 2758, US 3277, and US 3948) and four animals (US 2515A, US 2627, US 2780, and US 2757), and analyzed three new animal samples (US 2515d, US 3950, and US 3178a), selected for their relevance to the present study. Details on the protocols for the collagen extraction and measurement of isotope ratios appear in S1 Text.

We used multiple linear regression with isotopic ratios as outcomes to test for a possible association between the presence of animal remains and differences in human isotopic values, with age, sex, and animal-remains presence as independent variables. Statistical analyses were carried out in JMP statistical software (JMP®, Version 17. SAS Institute Inc., Cary, NC, 1989–2023) setting alpha at 0.05.

## Results

### Zooarchaeological, anthropological, and funerary patterns

The "human-animal co-burial" group (Table 1; S1A Table) includes four burials: B46 with US 2731 (female, 36–50 years old), B19 with US 2758 (full-term perinate), B102 with US 3277 (male, 36–50 years old), and B154 with US 3948, (male, 20–35 years old).

**Table 1:** Zooarchaeological and anthropological features of the human-animal co-burials (highlighted by asterisks) and the burials with animals as funerary food offerings of SV.

Burial	US Animal	Taxon	Animal skeletal remains	US Human	Anthropological sex (age at death)
B19*	2757	*Canis lupus familiaris*	complete skeleton	2758	NA(38 w)
B46*	2515a	*Equus caballus*	complete skeleton	2731; 2515f	F(36–50 y); NA (38 w)
B46*	2627	*Equus caballus*	R forelimb		
B46*	2791a	*Equus caballus*	ribs		
B46*	2515b	*Equus caballus*	mandible, maxilla		
B46*	2515c	*Equus caballus*	R coxal, teeth		
B46*	2780	*Canis lupus familiaris*	cranium		
B102*	3277/07	*Canis lupus familiaris*	complete skeleton	3277	M (36–50 y)
B154*	3950	*Equus caballus*	metapodial, mandible	3948	M (20–35 y)
B1	3159/01	*Sus scrofa domesticus*	L hind limb	3159	M (20–35 y)
B18	2729a	*Sus scrofa domesticus*	phalanges	2729	NA (34–36 w)
B18	2729b	NA	not determined		
B46	2515d	*Bos taurus*	radius	2731; 2515f	F (36–50 y); NA (38 w)
B46	2515e	*Caprinae*	femur		
B46	2791b	Herbivore	ribs		
B53	2603/04	*Sus scrofa domesticus*	L hind limb	2603	M (>50 y)
B84	2903	*Sus scrofa domesticus*	mandible, maxilla, ribs	2894	M (>50 y)
B97	3190/05	*Sus scrofa domesticus*	ribs	3190	NA (8–16 m)
B99	3251/06	*Caprinae*	coxal, femur	3251	M (20–35 y)
B100	3199/09	NA	not determined	skeleton absent	skeleton absent
B115	3212/06	NA	vertebra	3212	NA (6–9 m)
B131	3267/03	*Sus scrofa domesticus*	ribs	3267	F (36–50 y)
B147	3989/09	*Gallus gallus domesticus*	complete skeleton	3989	F (>50 y)
B148	3231/10	*Sus scrofa domesticus*	ribs	3231	M (20–35 y)
B147	3987a-b	*Pomatias elegans-Cepaea nemoralis*	shell		
B117	3178a	*Sus scrofa domesticus*	L hind limb	3178	NA (2–3 m)

US: stratigraphic unit, R: right; L: left; NA: not assessable, F: female, M: male, y: years, m: months, w: weeks.

**Fig 2:**
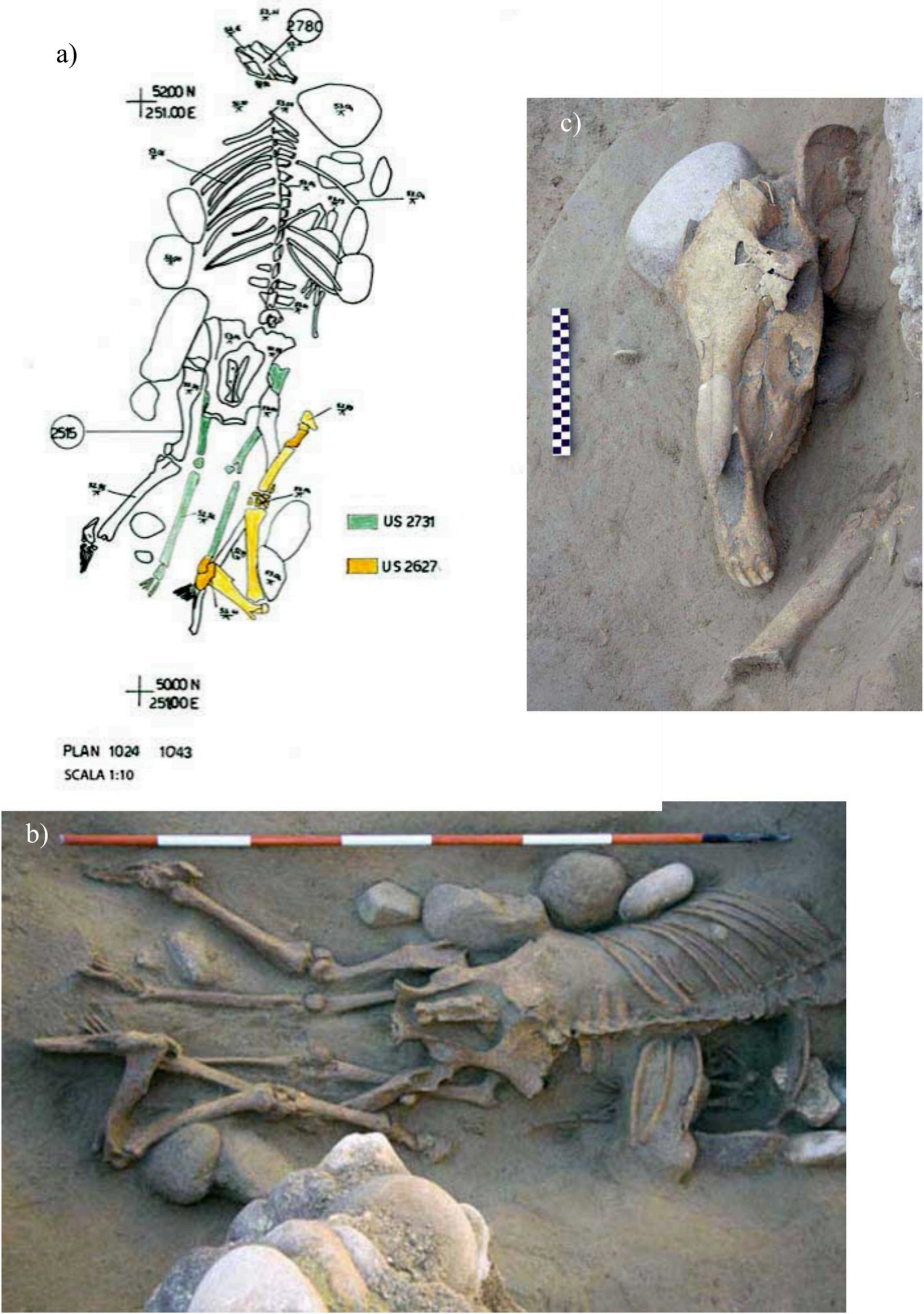
Plan (a) and picture (b) of B46, the burial of an adult woman (US 2731) associated with US 2515a (prone and articulated horse), US 2627 (horse forelimb), and US 2780 (dog cranium). The burial also contained the skeletal remains of three additional horses (not shown); (c) the cranium of US 2515a during excavation. (Photos by S.R.Thompson, courtesy of SABAP-VR Soprintendenza archeologia, belle arti e paesaggio per le province di Verona, Rovigo e Vicenza).

**Fig 3:**
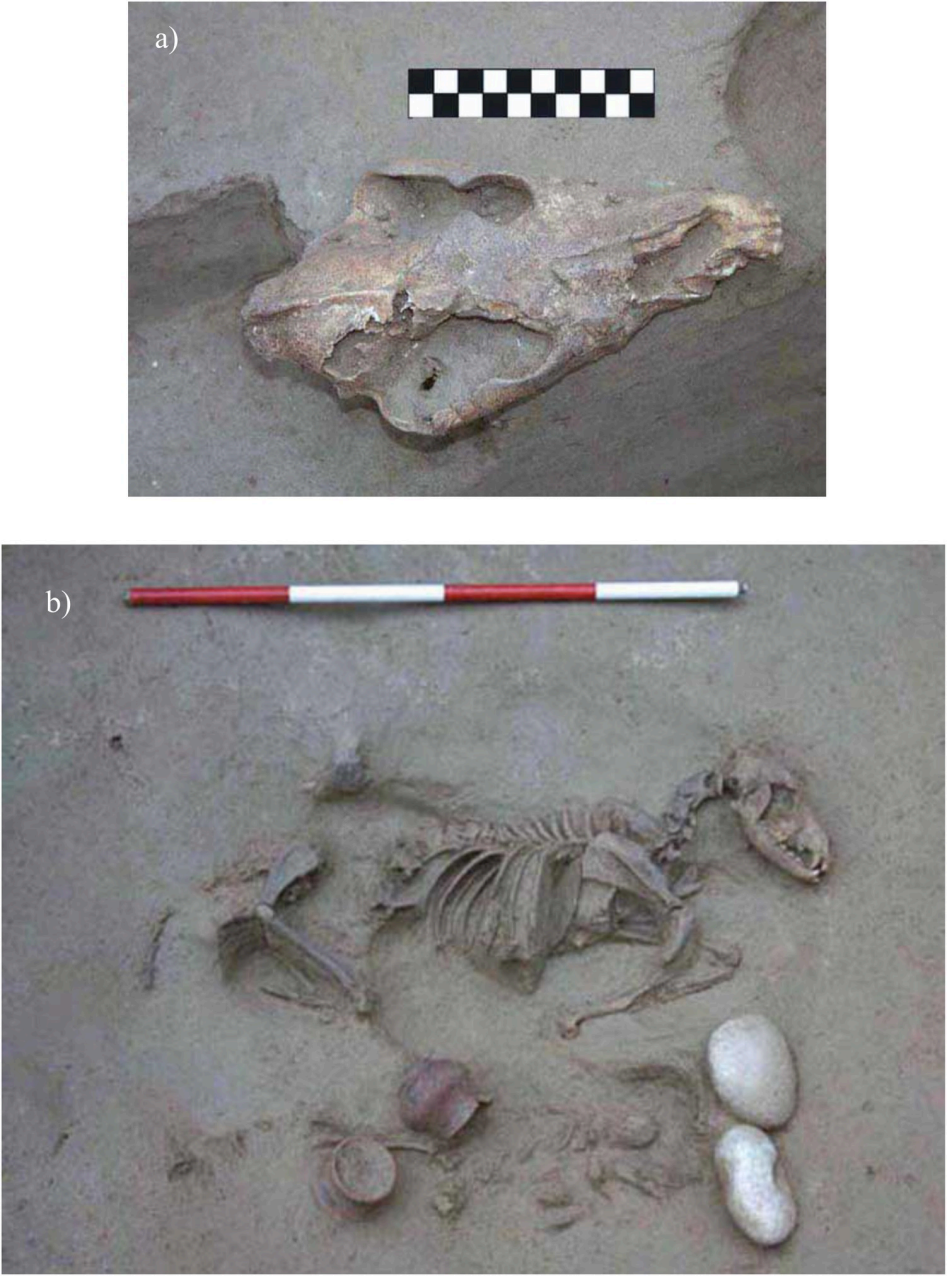
Dog cranium from B46 (a), and B19, joint interment of a dog and a human perinate (b). (Photos by S.R.Thompson, courtesy of SABAP-VR Soprintendenza archeologia, belle arti e paesaggio per le province di Verona, Rovigo e Vicenza).

The female, US 2731, was in a supine and extended position oriented north-east-south- west. Her grave goods consisted of two rings and a rich variety of faunal remains (Fig 2A) including: a near-complete horse skeleton (US 2515a) (Fig 2B and 2C), the articulated right forelimb from a second horse (US 2627), the remains of three additional horses (a few isolated horse ribs (US 2791a), mandibular and maxillary fragments (US 2515b), and a right coxal bone (US 2515c)) (Figs 2 and 3), a dog cranium (US 2780) (Fig 3A), bovid remains possibly related to food offerings (US 2515d), and the tooth of a goat or sheep (US 2515e) (S1 Table).

In addition to several carious lesions and strong dental wear, US 2731 exhibited signs of osteoarthritis in the cervical vertebrae (atlas-axis) and the coxo-femoral joints. Healed fractures are also present on two metatarsals of her left foot. The post-excavation zooarchaeological analysis identified the presence of a second, non-adult individual (US 2515f) whose remains were originally mixed with the faunal elements. Available morphological features (length of long bones, width and length of the left petrous part of the temporal bone [71] as well as the developmental stage of maxillary deciduous dental germs [70, 75]) point to an estimated age at death of around 36–40 weeks in utero. The most complete horse skeleton (US 2515a) from B46 was found directly above the woman and within the space defined by the stone structure lining the edges of the pit, with all features suggesting a simultaneous deposition of the animal and the woman. The horse was prone, oriented north-south with the head to the north, the hind limbs extended and partially abducted, and the vertebral column slightly flexed (Fig 2). The original anatomical connections were preserved with the exception of the head and forelimbs. The presence of an ancient wall (US 1273) to the north of the burial suggests that the disturbance to the anterior part of the skeleton could at least partly be due to the construction of this structure in antiquity. However, the position of the skull to the northwest and outside the edges of the burial, raises the possibility of its displacement already at the time of the original deposition. The heavily fragmented skull of equidae US 2515a can be classified morphologically as a male and dental wear suggests an age range of 10–17 years. The degree of fusion of postcranial epiphyses supports this estimate, pointing to an age >3 years. Radial length points to a withers height of 133 cm.

The morphological features of US 2627 (the right forelimb, vertebral elements, and rib fragments of a second horse) suggest an animal older than 18 months whose sex was not assessable and, based on metacarpal length, had a withers height of 129 cm.

Next to the remains of the two horses, B46 returned the isolated cranium of an adult dog (US 2780) (Fig 3A) exposed upon the demolition of the wall US 1273. Oriented east-west, the cranium was placed 75 cm to the north of the woman’s skull and at the same level as the head of horse US 2515a. This heavily fragmented dog cranium has bilateral preservation of the last three premolars (P2-4) and both molars (M1-2), pointing to an adult age at death, though the sex was not assessable. The stratigraphic relationships between the dog cranium, horse remains, and human inhumation suggest their contemporaneous deposition.

Additional faunal finds from this grave are radial and femoral elements from a bovid (US 2515d) and a *caprinae* (US 2515e) and rib fragments belonging to a large herbivore (US 2791b) (S1B Table).

**Fig 4:**
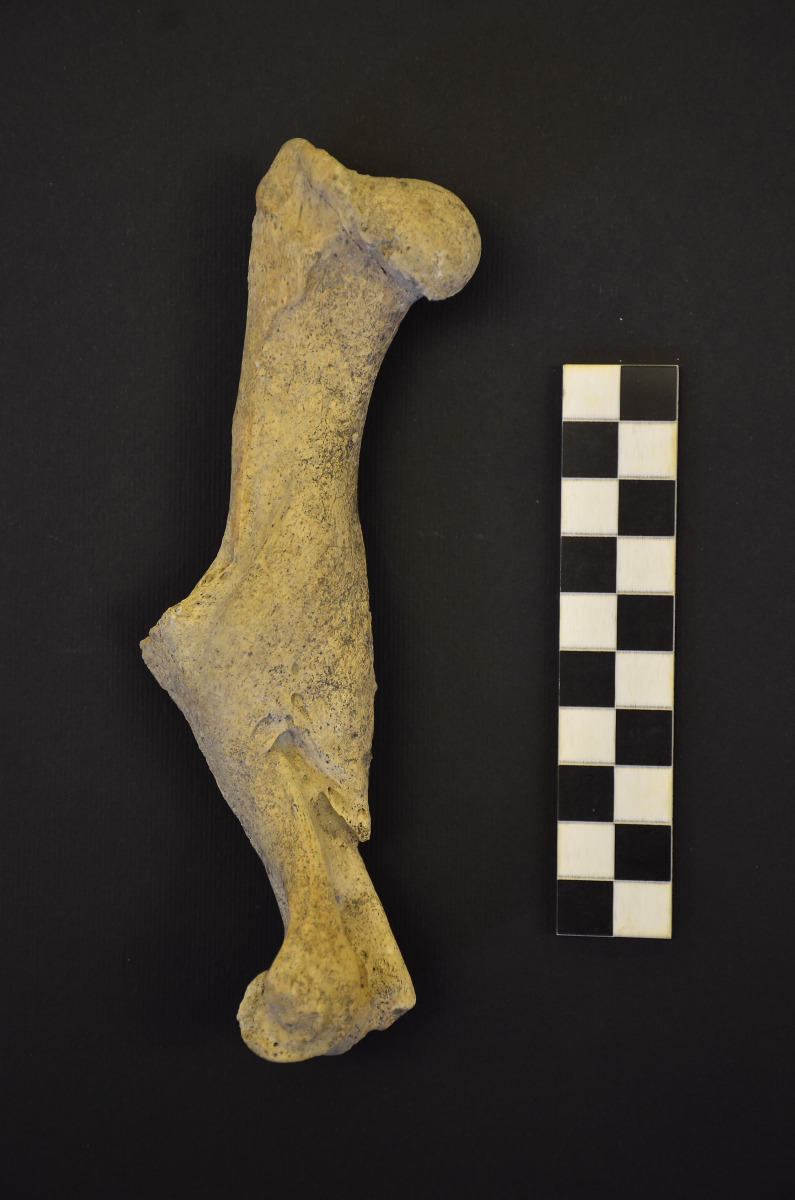
B19, US 2757: Healed fracture on the left humerus of the dog. (Photo by U. Tecchiati).

The second human-animal co-burial (B19) included a 38-week-old perinate (US 2758), the partially articulated skeleton of a dog (US 2757, Fig 3B), and two ceramic vessels (*ollae*). The extremely poor preservation of the human remains prevented the collection of possible paleopathological data. The animal was placed on its left side a few centimeters to the right of the perinate and along the same orientation (north-east-south-west with the head to the north-east). The dog skeleton is almost complete and overall well preserved and shows dental and skeletal features consistent with an age at death shortly after 18 months, however the sex is undetermined. Radial length points to a withers height of ca. 56 cm and there is a healed displaced fracture on the left humerus (Fig 4).

The third case of a human-animal co-burial (B102) is that of a middle adult male (US 3277) buried with a rich set of grave goods (17 elements including one knife and two finger rings) as well as a small dog (US 3277/07, sex unknown) whose scattered remains were found among pottery elements near the cephalic area of the burial space. The human individual was placed supine, extended, and oriented northeast-southwest (head to the northeast, facing west) and presented linear enamel hypoplasia in addition to periodontal and dental disease (carious lesions and abscesses) and an overall high degree of dental wear. He also demonstrated diffuse degenerative processes on the vertebral column (e.g. osteoarthritis and Schmorl’s nodes) and the left shoulder joint. A healed remodeled fracture was also documented on his 8^th^ left rib.

The last case of joint human-animal deposition is B154 containing US 3948, a young adult male placed supine and extended, oriented north-south (head to the north), and lacking grave goods. This individual was affected by periodontal disease and severe dental afflictions (carious lesions, abscesses, and the presence of calculus) and he presented nonspecific periosteal lesions on both tibiae, a greenstick fracture on his left clavicle, and a healed fracture on his 6^th^ left rib. A metapodial and mandible of an adult horse (US 3950), whose sex was not assessable, were placed above the left foot. While this burial revealed only two skeletal elements, the fact that they belonged to a horse led to its classification as a "human-animal co-burial". This decision is additionally supported by the distinct funerary appearance of horses not only at SV, but also in other similar contexts, such as in Transalpine Gaul [cf. 93]).

The second and largest group of zooarchaeological remains from SV are those pertaining to food offerings. S2 Text and Table 1 and S1B Table provide a detailed description of these finds. Here, we present a synthesis of the main zooarchaeological and anthropological patterns. There were a total of 16 food offerings from a total of 13 burials (one of which, B46, was also a human-animal co-burial). Pigs (*Sus scrofa domesticus*) are the most represented taxon (n = 8) followed by *caprinae* (n = 2), chicken (*Gallus gallus domesticus*, n = 1), bovid (*Bos taurus*, n = 1), an unidentified herbivore (n = 1), and other unidentified animal remains (n = 3). Two terrestrial snails from Burial 147 are not considered due to their possible intrusive origin (see S2 Text and S1 Table). With the exception of Burial 100, whose skeletal remains were likely removed during the construction of a later structure (see S2 Text), all burials contained human remains available for study. These include ten adults and six non-adults. The adult sample is composed of four male individuals aged between 20–35 years, three between 36–50 years (2 females and 1 male), and three older than 50 years (1 female, 2 males). The non-adults include three perinatal individuals and three infants aged within the first year of life.

### Human ancient DNA

Paleogenetic analyses of the human samples from SV were performed on 15 of the 16 individuals associated with animal remains, either as a co-burial or as a food offering (Table 2). The perinatal individual from B18 was excluded due to extremely poor bone preservation (cf. S1 Table). Three individuals (US 3948, US 3251, and US 3178, each of whom had food offerings) were successively excluded from downstream analyses as the genomic data retrieved did not meet our quality criteria. The remaining twelve individuals showed preservation of endogenous DNA (shotgun + enrichment) ranging from 16.16% to 66.13% and mean coverage of sequences mapping to the human reference genome between 0.17 X and 0.66 X. All samples show a typical damage pattern for aDNA (average deamination at 5´) (S1 Fig) and low contamination from modern human DNA at a nuclear (≤ 3%) and mtDNA (≤ 5%) level (see S2 and S3 Tables). The analyses revealed a total of 5 males (XY) and 7 females (XX) (S2 Table). Individuals US 2731, US 2758, and US 2515f were all confirmed to be females (XX) (see Table 2 and S4 Table). Moreover, the genetic analysis allowed us to rectify a morphological estimate that had classified individual US 2603 as male (the genetic tests yielded an XX result).

**Table 2:** Overview of the genetic results of the analyzed humans from SV. The asterisks highlight the human-animal co-burials. The “Human endogenous content (%)” values represent the percentage of human reads that mapped to the human reference genome. US: stratigraphic unit, YA: young adults (20–35 years old); MA: middle adults (36–50 years old); OA: old adults (> 50 years old); m: months, w: weeks.

Human Individual (US)	Age	Genetic Sex	Human endogenous content (%)	mtDNA Haplogroup	Y-Chromosomal Haplogroup
US 2731*	MA	XX	25.64487	H4a1c1a	-
US 2758*	38 w	XX	16.16487	T1a1	-
US 3277*	MA	XY	51.28234	U5b3	R1b1a1b1a2a1
US 2515f*	38–40 w in utero	XX	66.13427	H13b1+200	-
US 2603	OA	XX	50.37161	U5b1b2	-
US 2894	OA	XY	24.13820	J1c3	I2a1b1a1b1~
US 3159	YA	XY	26.67838	K1a+195	R1b1a1b1a1a2b
US 3190	8–16 m	XY	43.18187	U5a1a2b	R1b1a1b1a1a2c1
US 3212	6–9 m	XX	29.84288	T2h2	-
US 3231	YA	XY	45.47634	X2b6	R1b1a1b1a1a2b1
US 3267	MA	XX	27.77728	U5b1e1	-
US 3989	OA	XX	44.45667	H6a1b2	-

We then investigated kinship patterns among the studied individuals. Our results clearly show (using three different methods) that they were not related, at least up to the third degree, which is the maximum level that can be detected by the applied methods (S2 and S5 Tables). These data were further supported by the analyses of the uni-parentally transmitted markers (Y-chromosome and mitochondrial DNA), which are exclusively inherited from only one parent. In fact, all males carried different paternal Y-chromosome lineages (Table 2), even if it is possible that individual US 3159 is missing SNPs that define more derived R sub-lineages (as with US 3231) due to the absence of reads at this position or for low-quality data, therefore we cannot exclude that these individuals carried the same haplogroup. Moreover, all individuals (males and females) unambiguously carried different maternal mtDNA haplogroups (Table 2 and S2 Table). Overall, these findings strongly suggest no close biological relatedness among the individuals associated with both animal depositions and food offerings.

### Radiocarbon (^14^C) and stable isotopes (δ^13^C and δ^15^N) results

The ^14^C dating of the female adult individual from B46 (US 2731: BE-18889.1.1) is 351–52 cal. BCE, the full-term perinate US 2758 (BE-18890.1.1) from B19 is 198–46 cal. BCE, and individual US 3277 (BE-18895.1.1) from B102 is 353–54 cal. BCE (see S6 Table). The radiocarbon results of these individuals are thus consistent with the relative chronology (3^rd^-1^st^ c. BCE) proposed for SV after a preliminary study of the archaeological record.

**Fig 5:**
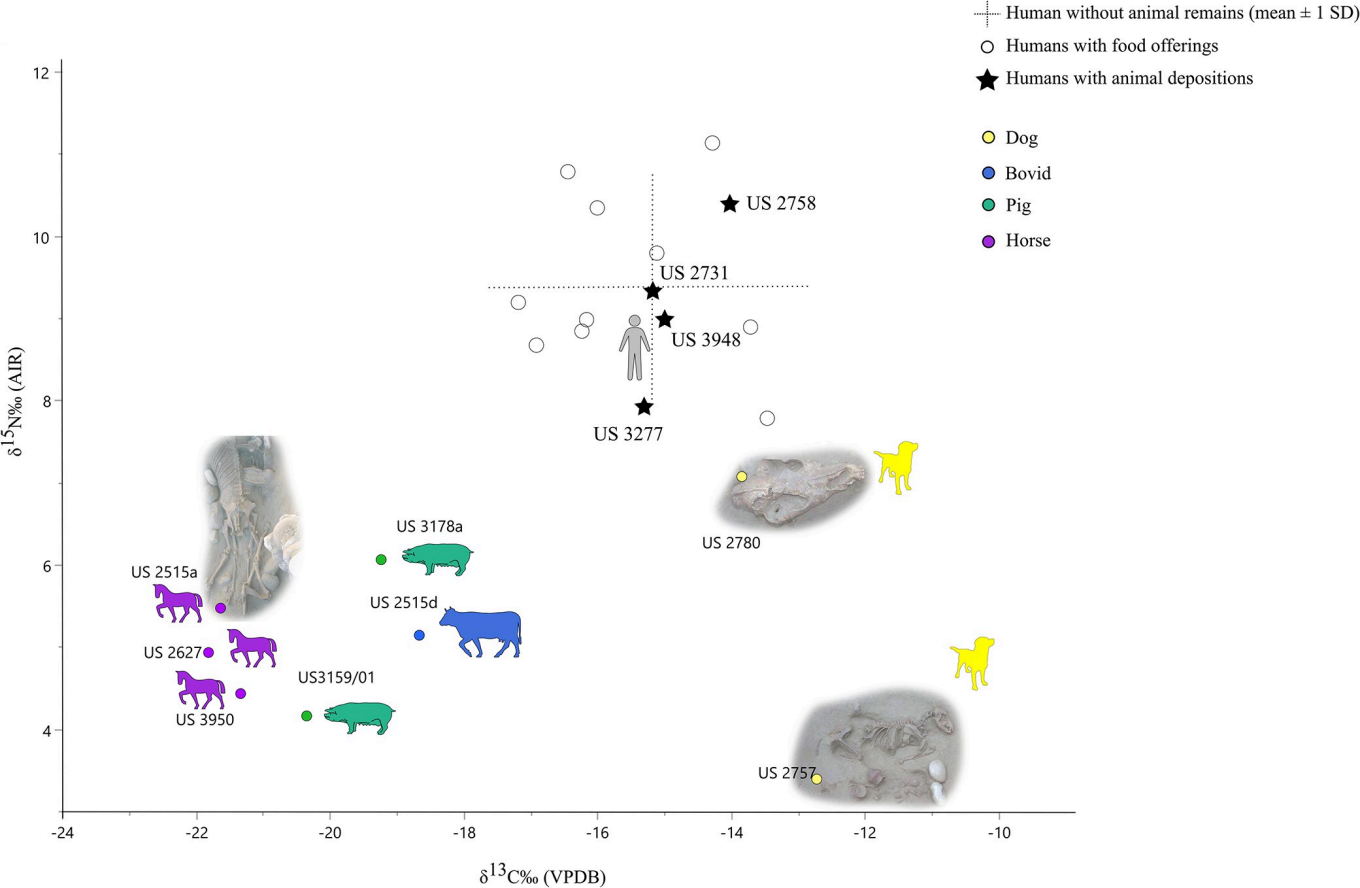
Carbon and nitrogen isotopic ratios of humans and animals from SV. The human range is based on the mean ± 1 SD of individuals without food offerings/associated animal depositions. Humans with food offerings and associated animal depositions are plotted separately for comparison as empty circles and stars respectively. The isotopic data of US 3159/01 have been already published in [42].

Fig 5 shows the isotopic values (δ^13^C and δ^15^N) of the analyzed faunal specimens while Table 3 presents the isotopic range of humans with and without associated faunal remains (both co-burials and burials with food offerings).

**Table 3:** Isotopic ranges of humans buried with and without animal remains. N: number of samples; SD: standard deviation.

	No animal remains						With animal remains					
	δ^15^N‰ (AIR)			δ^13^C‰ (VPDB)			δ^15^N‰ (AIR)			δ^13^C‰ (VPDB)		
	N	Mean	SD	N	Mean	SD	N	Mean	SD	N	Mean	SD
All adults	45	8.8	0.7	45	-15.2	2.1	10	8.9	0.6	10	-15.44	1.2
F (adults)	19	8.5	0.8	19	-14.5	2.0	4	8.7	0.7	4	-15.3	1.3
M (adults)	26	9.0	0.6	26	-15.7	2.1	6	8.9	0.6	6	-15.6	1.3
Non adults	33	10.1	1.6	33	-15.1	2.6	4	10.7	0.4	4	-15.2	1.2

Stable carbon and nitrogen isotope values of the new human (n = 4) and animal (n = 7) individuals of SV included in this study are reported in Table 4.

**Table 4:** Human and animal stable isotope values (δ^13^C and δ^15^N) and relative quality criteria.

Individual	Species	Bone	δ^13^C‰(VPDB)	δ^15^N‰(AIR)	%C	%N	C/N	% coll. yield
US 2731	human	cranium	-15.2	9.3	41.3	15.1	3.2	3.2
US 2758	human	femur	-14	10.4	37.1	13.7	3.2	5.7
US 3277	human	cranium	-15.3	7.9	43.5	16.1	3.1	6.1
US 3948	human	cranium	-15.6	8.7	41.2	15	3.2	2.5
US 2515a	horse	R metatarsal	-21.6	5.5	42.8	15.8	3.2	2.8
US 2627	horse	R metacarpal	-21.8	4.9	40.1	14.9	3.1	1.8
US 2515d	bovid	R femur	-18.7	5.1	41.1	16.3	2.9	2.9
US 2780	dog	cranium	-13.8	7.1	41.4	15.6	3.1	2.3
US 2757	dog	metatarsal	-12.7	3.4	39.3	14.6	3.1	2.3
US 3950	horse	mandible	-21.3	4.4	35.5	12.9	3.2	1.8
US 3178a	pig	carpal bone	-19.2	6.1	42.3	15.6	3.2	6

**Fig 6:**
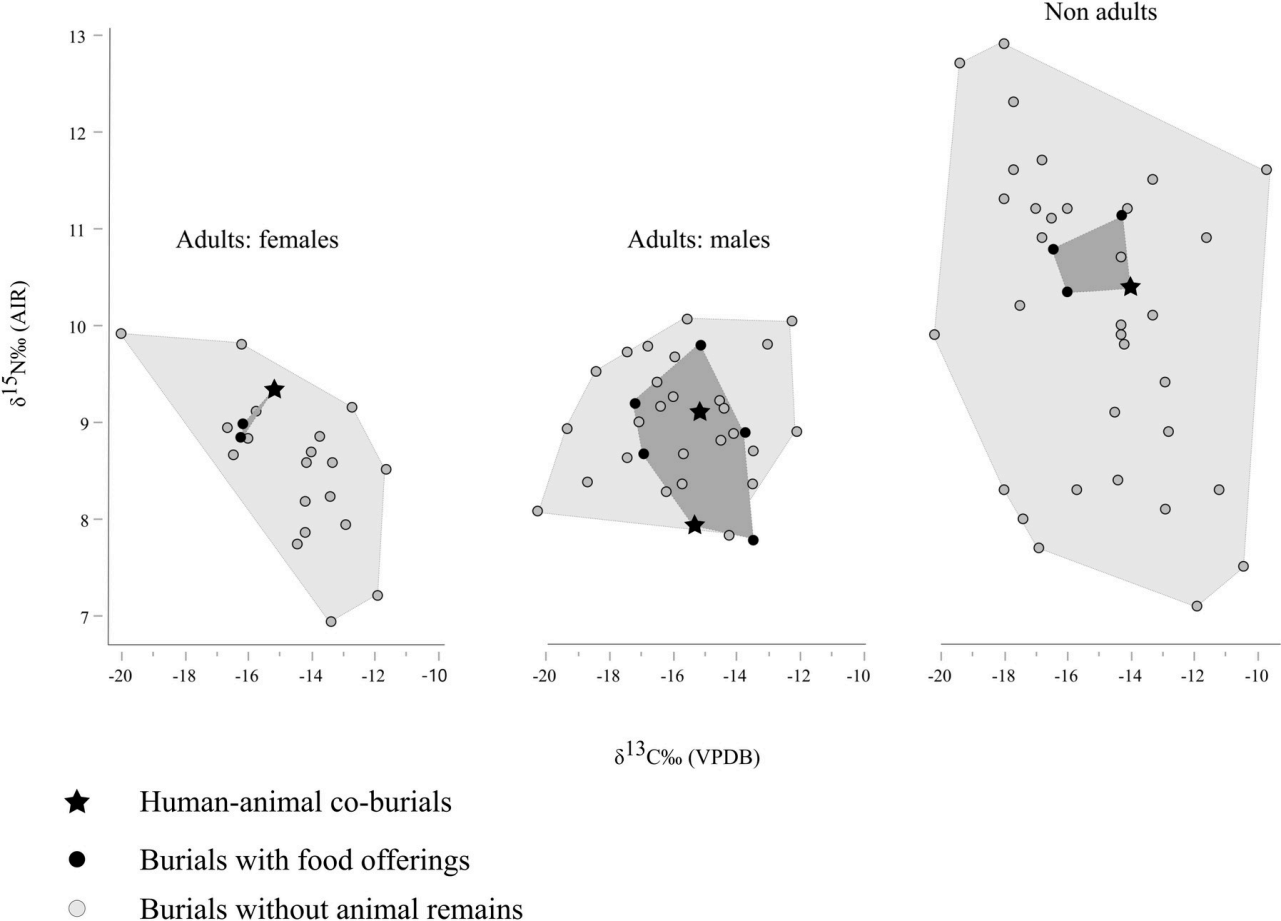
Carbon and nitrogen isotopic ratios of humans without (light grey) and with (black) associated animal remains. Black stars indicate human-animal co-burials.

Individuals accompanied by animal remains (as food offerings or in the context of human-animal co-burials) fall in the isotopic range of the other humans from SV without showing any specific trend (Fig 6).

This is further confirmed by the multiple regression, which highlights a lack of statistically significant association between carbon and nitrogen isotopic values and the presence of animal remains, including when controlled for age and sex (Table 5).

**Table 5:** Results of the multiple regression performed to test the association between isotopic values, age, sex, and presence of animal remains in burials. (a) All humans; (b) adults only. NP: number of parameters; SS: sum of the squares; DF: degrees of freedom. Bold characters highlight statistically significant associations.

a) all humans	δ^13^C‰ (VPDB)					δ^15^N‰ (AIR)		
	NP	DF	SS	F Ratio	p	SS	F Ratio	p
Age class	5	5	11.09	0.44	0.823	56.15	10.48	**<0.0001**
Presence of animal remains	1	1	0.15	0.03	0.863	0.29	0.27	0.602
b)adults only	δ^13^C‰ (VPDB)					δ^15^N‰ (AIR)		
	NP	DF	SS	F Ratio	p	SS	F Ratio	p
Sex	1	1	15.18	3.90	0.054	2.05	5.12	**0.028**
Age class	1	1	0.26	0.07	0.799	2.85	7.09	**0.010**
Presence of animal remains	1	1	0.42	0.11	0.745	0.07	0.19	0.668

When focusing on the isotopic values of horses and dogs, a clustering of the former toward lower values was noted (averages for carbon and nitrogen of -21.59 ± 0.24‰ VPDB and 4.94 ± 0.52 ‰ AIR respectively). The two dogs show relatively higher carbon ratios (mean and SD of -13.28 ± 0.79 ‰ VPDB), which fall in the upper range of humans. Conversely, they sharply differ for their nitrogen ratios. US 2780 (isolated cranium) presents a δ^15^N of 7.1‰, a value consistent with the expected omnivorous diet of this species. US 2757, the dog buried near the female perinatal individual, shows an extremely low value (3.4‰ AIR), lower than that of herbivores (Fig 5).

## Discussion

### Types of finds and their possible meaning

The main question of this study is the possible social meaning of the few human burials with animal depositions and food offerings at SV. Even considering the archaeological invisibility of possible soft tissue offerings (cf. [9]), the low number of burials returning complete or fragmentary zooarchaeological remains (16/161: 9.9%) is nonetheless interesting. Table 1 and S1 Table summarize the lack of demographic or funerary patterns among the burials with animals, a possible exception being the fact that both inhumations accompanied by fully preserved animals (US 2731, middle adult, and US 2758, full-term perinate) are female. This result, highlighted by aDNA analyses, is tantalizing and may (cautiously, given such small number) suggest some kind of specific ritual reserved for females. On the other hand, we do not know if there was already a social identification of gender roles for perinatal individuals. However, what we have been able to observe is that US 2758 was buried with grave goods, among which was a pyx (a small ceramic cylindrical box with lid, often used for cosmetics and toiletries) with two graffiti in the Lepontic alphabet. Unfortunately, only the first letter (an "a") of the graffiti was readable. It is therefore unclear if the writings referred to a name or to something else [50, 51]. The isotopic values of individuals associated with animal remains suggest that their diet did not differ substantially from that of the rest of the population (at least in terms of access to animal proteins and C_3_/C_4_ plant products). Additionally, previous paleopathological data on SV [38, 39, 43] did not highlight any significant association with funerary variability, pointing to a homogenous exposure in this population to developmental stressors. Moreover, aDNA data point to a lack of biological relatedness up to the third degree between these individuals, which would in turn exclude a link between animal depositions, close kin groups, a possibly inherited status, and family/clan-based mortuary traditions, though biological relatedness on a broader scale cannot be excluded. This result represents the first paleogenetic analysis of this funerary context; however, it will need to be better contextualized through comparisons of kinship patterns and funerary variability with a larger sample, as seen in recent studies [e.g., 94, 95].

While the anthropological, funerary, isotopic, and paleogenetic data do not imply a simple explanation for animal depositions and food offerings at SV, the cultural basis of these practices may reside in the interplay between diverse variables. These factors encompass the various components of individual status (e.g., social, economic, and acquired), affective factors (e.g., pets accompanying their owner), subtle changes in rituals over time, the influence of exotic customs, and the presence of nonlocals within this community [41, 96]. Furthermore, it is important to stress two well-known issues affecting any funerary archaeological study like ours. First, what we observe is only a fraction of the material aspects of the ritual. In addition to the inevitable loss of information due to the degradation of organic materials, the observed remnants provide only approximations, lacking crucial components of ritualistic performances like sensorial and gestural elements. Second, funerary rituals are communal representations enacted by the surviving community members, shaped by their shared needs and symbolic codes. Accordingly, interpretations of burials, including those from SV, need to take into account their link to the deceased social persona [97] and biography [98] as well as how the survivors mediated, interpreted, and potentially imbued additional meanings into the death and circumstances surrounding it.

### Human-animal co-burials: Horses and dogs in La Tène symbolism

Among the finds from SV, those of horses and dogs deserve particular attention due to their deposition as both articulated and isolated remains, their co-presence in at least one burial, and the apparent relevance of these animals in La Tène symbolism.

#### Horses

As mentioned in the introduction, the deposition of horses alongside humans is well-known in the archaeological record. While this is likely driven by heterogenous (and difficult to ascertain) cultural factors, it nonetheless reflects the widespread social and economic centrality of this animal in human communities both past and present (e.g. burials of horses and humans in modern Tuvan society, see [99].

Turning to SV, our finds substantially expand the knowledge of horse deposition in Cenomane funerary contexts, which exhibit similarities with the same practice among the Senones. This convergence suggests shared ideologies among the Transalpine groups [93] and emphasizes the importance of horses as symbols of a social status not necessarily limited to males. This would explain the association between this animal and the female inhumation US 2731. Vitali [11] describes other two cases of joint horse-human female burials, one among the Senones of Montefortino d’Arcevia and the other among the Cenomani of Carzaghetto di Canneto sull’Oglio. In the first context, dated between the end of 4^th^ and beginning of the 2^nd^ c. BCE, the archaeologists reported a double burial of a horse and woman in which the horse lay over the grave of the woman and was associated with pottery and elements of a harness. In the case from Carzaghetto (Mantova, 4^th^ -3^rd^ c. BCE; [37, 100]), a horse grave was located near the inhumation of a probable female individual, although doubts pertain as to the actual relationship between these two burials [11]. Similarly to SV, this is a unique finding among the 56 burials identified in this necropolis and together with the fragmentary archaeological evidence available from Italy, it strongly hints at a deep symbolism attributed to horses in these “Celtic” cultures. This is additionally supported by imagery of the goddess Epona, whose name derives from the Celtic word for horse (*epos*–[101]) and whose iconography is regularly characterized by the death of one or more of these animals. A divinity largely worshipped among Gallo-Romans, Epona held a complex symbolism linked to fertility and protection of the individual after death ([102]: 204–207; [103]). An association between the horse remains at SV and the role of Epona as a guide who accompanies newly deceased souls to the afterlife (psychopompic nature) is therefore an interesting possibility. The ritual importance of horses is further suggested by the relative frequency of this animal’s remains from other La Tène contexts outside of Italy [21–24, 104, 105], where bones may present traces of manipulation similar to those observed on the humans remains [9]. Besides these religious considerations, we may also ask if the presence of horses in burials was somehow related to the practice of horse riding by the deceased during life. Indeed, the individuals from SV buried with horses do show intriguing paleopathological evidence of this. For example, the presence of healed fractures in the upper limbs (US 3948), ribs (US 3277 and US 3948) and lower limbs (US 2731) and the diffuse degenerative processes of the vertebral column and limbs (US 3277, US 2731) may cautiously suggest this type of practice, as well as the associated risks, e.g. falls [106–109].

#### Dogs

Dogs at SV seem to share a comparable ritual relevance with horses, the only difference being that horses, similarly to what was observed in Gaul [9, 25, 93], are also represented by isolated limbs or limb elements. This similarity fits the apparent special symbolic meaning of dogs and horses in "Celtic" cultures highlighted by some authors and signaled archaeologically by the co-occurrence of these animals as funerary offerings [9, 110]. Based on the available data, we cannot offer a specific interpretation of the presence of dogs at SV. Dogs were occasionally consumed in Gaul and other La Tène contexts during the Late Iron Age, as evidenced by the presence of butchering marks on canid skeletal remains found in settlements and sanctuaries (e.g., [111]). Also, they are assumed to have been used as funerary food offerings [112]. At SV, the anatomical representation of dog remains (one cranium and two complete skeletons) and the lack of butchering marks do not support their ritual consumption nor their use as food offerings. Rather, and especially in the case of the two preserved skeletons, they suggest that the animal was sacrificed, possibly due to a precise symbolism associated with dogs in this culture. In particular, it is tempting to cautiously postulate an association between the deposition of this animal and their association to the underworld. A link between dogs and the afterlife can be found throughout time and space, with examples from ancient Egypt, Scandinavia, Classical, and Gallo-Roman cultures [113–115]. This association between dogs, death, and the afterlife has also survived in some Southern Italian regions (Sicily, Apulia, Basilicata, and Campania–[116]).

The typological and radiocarbon dating of SV place the use of this necropolis during, but probably also preceding, the Romanization phase. A reading of these funerary rituals exclusively through a "Celtic" interpretive lens may therefore lead to dangerous oversimplifications. Rather, we need to consider the possibility of a "creolized" negotiation between local and classical cultural identities (cf. [117]), as further suggested by the co-presence among the artifacts retrieved at SV of Transalpine and Roman typological elements. In Ancient Greece, dog sacrifices were dedicated to the earth goddess Eiloneia (or Eilethyia) who watched over births and childhood development, and this divinity is also associated with Hecate and the Phoenician goddess Astarte [114]. In Gallic contexts, female deities linked to birth, regeneration, and growth (such as Sirona, Nehalennia Aveta, or the already mentioned Epona) are often depicted with a small dog on their lap or crouching at their feet [118]. The funerary association of women and perinates with dogs can also be linked to the cult of the Roman goddess Genita Mana, an Italic divinity who presided over the menstrual cycle and related to the sphere of fertility [114]. According to Pliny (*Naturalis Historia*, 29, 58) and Plutarch (*Quaestiones Romanae*, 52, 277), the Greeks sacrificed a female dog to Hecate, while Romans offered the same sacrifice to Genita Mana when someone was born. Human-dog co-burials, especially in association of women and perinates, could therefore be related to rituals of purification performed after the death of an infant to prevent future miscarriages or premature deaths [114, 119]. Examples of human-dog co-burials involving females and/or perinates or infants have also been documented in the Greek archaeological record (e.g., Grave 16 at Areopagus, the well deposit of Kolonos Agoraios in Athens dated in the 2^nd^ century BCE, and at the Agora of Messene of the 3^rd^ century BCE) [120–123] as well as in the Italian and Iberian peninsula during Roman times (e.g. the necropolis of Lugnano in Teverina, Italy dated to the 5^th^ c. AD, the Roman town of Peltuinum in central Italy dated between the 1^st^ c. BCE and the 5^th^ c. AD, and the burial site of Llanos del Pretorio, Córdoba, Spain dated to the 1^st^ c. AD) [115, 124, 125]. It is also worth mentioning the unique case of the Late Iron Age rural settlement of Gamsen (Valais, western Alps, Switzerland), which is culturally and chronologically close to SV (La Tène D2, c. 80–30 c. BCE). Here, archaeologists uncovered four neonate burials associated with the complete skeletons of a female dog and a male piglet. These findings were interpreted as a funerary rather than a foundational ritual [126]. One last explanation for the human-animal co-burials of SV is that the woman and perinate were simply buried with the domestic animals to which they were affectively associated. This does not exclude the possibility that certain animals were associated both with a specific symbolism in this culture as well as having been loved companions during life. This leads us to wonder if the presence of dogs (as well as horses) in burials may be related to their roles in hunting activities. The importance of dogs and/or horses as hunting companions among "Celtic" societies is suggested by classic authors (e.g. Arrian’s *Cynegetica*, III) and especially iconographic data. Hunting scenes depicting horses and/or dogs as well as prey appear on a variety of materials, including pots, figurines, sculptures [102: 49–50] and, famously, the rock art from Camonica Valley (northern Italy), mostly dated to the Iron Age and geographically close to SV [127, 128]. Intriguingly, a ritual link to hunting, has been also proposed as an explanation for faunal deposits from Britain and France featuring horses and dogs, as well as funerary contexts including the association between these animals [102: 56–57, and references therein]. A symbolic association between hunting and the presence of dogs and horses at SV is therefore an interesting, although explorative, hypothesis.

### Human-animal relationships

Isotopic data help explore the diet of domestic animals at SV and, in turn, develop hypotheses regarding management strategies of these animals by humans. Horse samples show similar isotopic values between each other and, in general, the lowest carbon isotopic ratios among the analyzed animals. This fits isotopic ranges for this taxon previously reported by other studies and interpreted based on environmental (cfr. Canopy effect- [129–132] and/or metabolic factors (e.g., [133]).

The two dogs exhibit interesting isotopic data with similar δ^13^C values but contrasting δ^15^N values. The carbon suggests that the diet of both dogs included C_4_ plant products. Previous isotopic analyses of SV interpreted the high carbon isotopic values of the human dataset as the result of a mixed diet incorporating millet [42], possibly in the form of porridge. Apparently, the same resource was also quite important for dogs, who likely accessed it via kitchen and food wastes or human feces. The δ^15^N bone collagen value of US 2780 (Burial 46) is consistent with that expected from an omnivorous diet incorporating both plants-cereals and animal proteins. Conversely, the other dog US 2757 (burial 19) shows an extremely low δ^15^N ratio, even lower than those of herbivores and strongly hinting at a vegetarian diet. When considering this result, it is also interesting that the δ^13^C bone collagen ratio of this animal is higher than that of the other dog US 2780. This may suggest that the lower access to animal proteins was somehow accompanied by a higher consumption of C_4_ food sources. To better contextualize these results, we can compare the difference in isotopic values between the dogs and the adult humans at SV with the same differences calculated by Guiry [134] based on published data from various geographical and chronological contexts. This comparative set shows that on average dogs exhibit slightly lower nitrogen isotopic values compared with humans from the same context (average dog-human difference: -1.16±1.3‰) (S2 Fig). Conversely, dog and human δ^13^C tend to be more similar (average dog-human difference: 0.19±0.8‰). When comparing the two dogs (US 2757 and US 2780) from SV with these tendencies, the low nitrogen isotopic value of US 2757 stands out even more, as well as the high carbon values for both of them. The dogs from SV are therefore an interesting example of different types of commensalism with humans. The specific diet of the dog with less access to animal proteins and a higher carbohydrate supply (C_4_ plants, millet) is unclear; however, we can propose two working hypotheses: (a) the dog was originally selected for being ritually sacrificed, and its specific and restricted diet was somehow linked to this special role [135], or (b) the diet of the animal was linked to its socio-economic role, which possibly demanded more strenuous physical activity (e.g., guarding herds, hunting) [132, 134, 136, 137] or as a food item or other socio-economic function (e.g., fur exploitation [138]). The healed fracture on the left humerus of US 2757 may be related to such a role and the associated exposure to biomechanical stressors. It is also possible, although in our case not demonstrable, that the different diet may be associated with the fact that the dog in question has suffered and recovered from this injury. However, it is necessary to note that humeral fractures are relatively common in dogs, especially in immature males, with reported frequencies reaching 8–14%, and mostly caused by falling from heights (and to a lesser extent to traffic accidents) (e.g., [139, 140]). Fractures of this type are not frequently reported in the zooarchaeological literature; however, a few examples are described in Roman literature (see [141]). However, whereas Roman veterinary sources highlight various remedies for different afflictions in livestock (e.g. bandaging for broken bones etc.), MacKinnon [141] postulates no human intervention in the case of the fractures observed in dog remains. The same hypothesis may apply for US 2757, especially based on the badly aligned and not correctly set fracture ends. However, this does not rule out other attitudes of care (e.g. a dedicated feeding and care of the animal) [141–144]. With regard to the origin of this lesion, the morphology of the fracture (oblique) points to an indirect trauma, e.g. falling from height [145].

Differences in bone growth and remodeling rates between humans and dogs can affect the resolution of isotopic data, with dogs being more sensitive to short-term dietary changes [134]. One possibility is therefore that the outlier position of US 2757 for nitrogen may actually be related to a brief deviation in the diet of this animal before death rather than to a continued, differential access to food sources. Another factor worth considering is caecotrophy (feces consumption), a typical habit in dogs [146, 147] which has been previously signaled as a possible influence on the isotopic ratios observed for this taxon [148]. Differences between fecal and dietary isotopic ratios have been indeed confirmed experimentally by a study [149] carried out on fecal samples from 14 human subjects fed controlled diets. In general, results highlighted lower δ^13^C values in feces compared with the overall diet. Conversely, different dietary regimens were reflected by different offsets for nitrogen isotopic ratios, with meat-based and vegetarian diets corresponding to lower fecal δ^15^N values and fish-based diet leading to higher nitrogen isotopic ratios [149]. The dog (US 2757) however shows an opposite trend—higher δ^13^C and lower δ^15^N values compared with those of humans—and this would signal no substantial effective role of caecotrophy in our case.

### Animals as funerary food offerings

Turning to the animal offerings, it seems worth highlighting a specific ritual now documented by different cases in the Cenomane cultural milieu, i.e., the funerary offering of pig hind limbs. This is observed not only at SV (e.g., burials 1, 53, and 117 among others) but also in another "Celtic" context from the same area and the same chronology, i.e., at Casona di Nogara in the province of Verona [150]. A previously unpublished case comes from the famous "baby prince" chariot tomb (T7) of Lazisetta di Santa Maria di Zevio (late 2^nd^ c. BCE), also located in the province of Verona [151]. In addition to the rib of a large herbivore, the faunal finds included various remains of pig hind limbs (see S7 and S8 Tables for details), in line with what has been observed in other La Tène contexts where pig food offerings include especially high-quality parts of the animal [110]. Also, other Cenomane contexts of the 2^nd^ and 1^st^ centuries BCE from the surrounding area of Verona (Casalandri Isola Rizza, Valeggio sul Mincio, Povegliano Veronese, Ortaia locality) highlighted a prevalent presence of pig offerings, followed by poultry (mainly hens) and in significantly smaller amounts sheep and freshwater fish (cyprinids) [152–155]. An interesting aspect highlighted by Méniel [152] for the context of Povegliano Veronese is the notable presence of very young animals and even fetuses, especially among pigs, which partly deviated from the typical tendency among the "Celtic" groups to include only fully grown animals in their funerary rituals. The same author proposes an intriguing parallelism between the age of the deceased and the presence of young animals; the necropolis of Povegliano Veronese shows a majority of non-adults and individuals in the perinatal age phase. This trend, on the other hand, is not reflected at SV even though the frequencies of non-adults and perinates [38] are very similar to those observed at Povegliano Veronese. Another quite interesting association documented at SV is the human-chicken co-burial (Burial 147) (S2 Text) in which the skeleton of the animal is almost complete and did not present traces of butchery. The funerary inclusion of complete chickens is diffused throughout northern Europe and Britain during the Late Iron Age and early Roman period [156, 157]. In British contexts, this practice seems to be differentiated between males and females, with the former being buried with cockerels and the latter with hens (e.g., Broughton, Yorkshire [158]). According to Best and colleagues [158], chickens could have been attributed to a psychopomp role (see e.g., Temple of Uley, Gloucestershire [159]). In other cases, the presence of chicken remains clearly served as food offerings, and one commonly suggested interpretation (also applicable to "Celtic" contexts located farther south of the Po plain in Italy (e.g., Monterenzio Vecchio, Monte Tamburino) links the presence of animal remains in burials with the ideology of the symposium and funerary banquets, during which animals were sacrificed and then consumed by the participants to honor the deceased [160].

## Conclusions

Funerary rituals are highly formalized and symbolically rich moments during which a community represents, reinforces, and transmits its cultural, social, and economic constitutive elements. Accordingly, and considering the cultural contingency of animal symbolism, archaeological discussions of animal remains from burials may benefit from the inclusion of various analytical and interpretive perspectives. Based on this consideration, we approached the study of the few animal funerary depositions in a Late Iron Age funerary context (the Cenomane burial site of Seminario Vescovile, Verona, Italy) in an attempt to connect and jointly discuss zooarchaeological, archaeological, anthropological, isotopic, and paleogenetic data. The combination of these different datasets allowed us to highlight some important trends, including: (a) the absence of dietary, genetic, demographic, and funerary similarities among burials containing animals as well as a lack of association between these mortuary practices and the straightforward notion of social status based on age, sex, and/or close biological kinship, (b) the consistency of these finds with Transalpine (La Tène) cultural traditions, possibly mixed with local (Venetic) and Mediterranean (Roman) elements, and (c) differing management strategies of dogs, possibly influenced by economic and/or ritual factors. These results, as well as the new questions raised by this analysis, stand as a case in point of the suitability of multiple interpretive angles when approaching the traces left by past funerary behaviors.

## Supporting information

S1 Table Summary table including zooarchaeological, anthropological and archaeological features of the human-animal co-burials (a) and the burials with animals as funerary food offerings (b). US: stratigraphic unit, R: right; L: left; NA: not assessable, F: female, M: male, y: years, m: months, w: weeks, NE: north-east, SW: south-west, NW: north-west, SE: south-east, N: north, S: south, XX: genetically female, XY: genetically male *contaminated library.
(XLSX)

S2 Table Overview of the results of paleogenetic analyses for SV samples. US: stratigraphic unit, NA: not assessable, F: female, M: male, y: years, m: months, w: weeks, * contaminated library.
(XLSX)

S3 Table Summary of mapping statistics, uniparental markers, genetic sex estimation, and damage statistics for SV samples. US: stratigraphic unit, NA: not assessable, F: female, M: male, y: years, m: months, w: weeks.
(XLSX)

S4 Table Summary of the laboratory work for the paleogenetic analyses for SV samples. (XLSX)

S5 Table Kinship estimated for SV samples. (XLSX)

S6 Table Radiocarbon results and quality data. BP: before present; 1sd: 1 standard deviation.
(XLSX)

S7 Table Osteometric measurements (mm) of pig remains from Lazisetta (after [49]). (XLSX)

S8 Table Osteometric measurements (mm) of animal remains from SV (after [49]). (XLSX)

S1 Fig Fragmisincorporation Plot (mapDamage 2.2.1 [67]). (TIF)

S2 Fig Differences between the isotopic values of dogs US 2780 and US 2757 and the adult humans from SV, plotted against published differences between dogs and human mean isotopic ratios from a series of worldwide contexts (data from [100]). (TIF)

S1 Text Methods. (DOCX)

S2 Text Details on burials with food offerings and additional zooarchaeological information. (DOCX)
