# Supplementary figures and images for: "Until death do us part". A multidisciplinary study on human- Animal co- burials from the Late Iron Age necropolis of Seminario Vescovile in Verona (Northern Italy, 3rd-1st c. BCE)
Source: PLoS One. 2024 Feb 14;19(2):e0293434. doi: 10.1371/journal.pone.0293434 (PMC10866530; doi:10.1371/journal.pone.0293434)

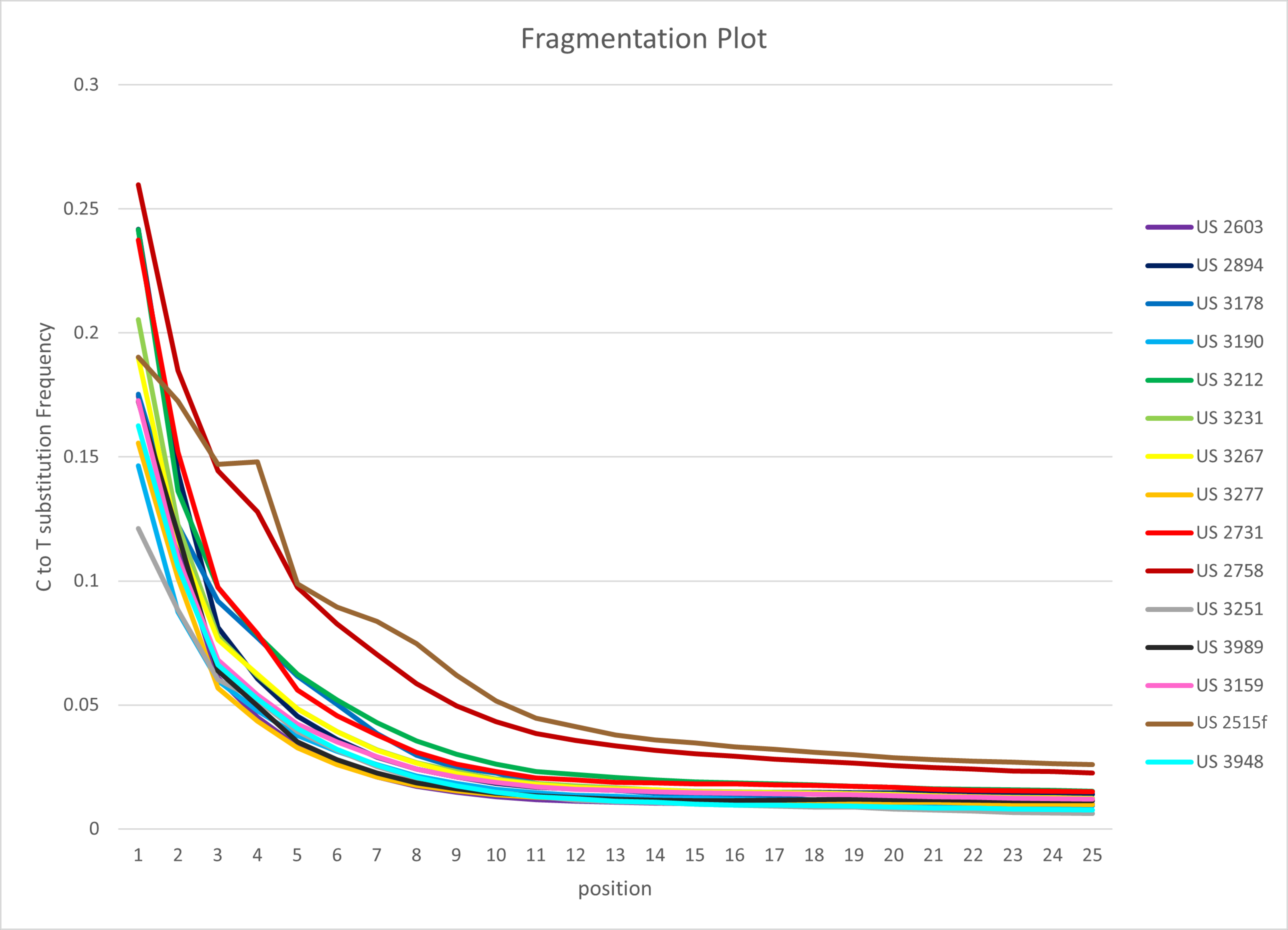

Supplement: S1 Fig — (TIF) [file pone.0293434.s009.tif]

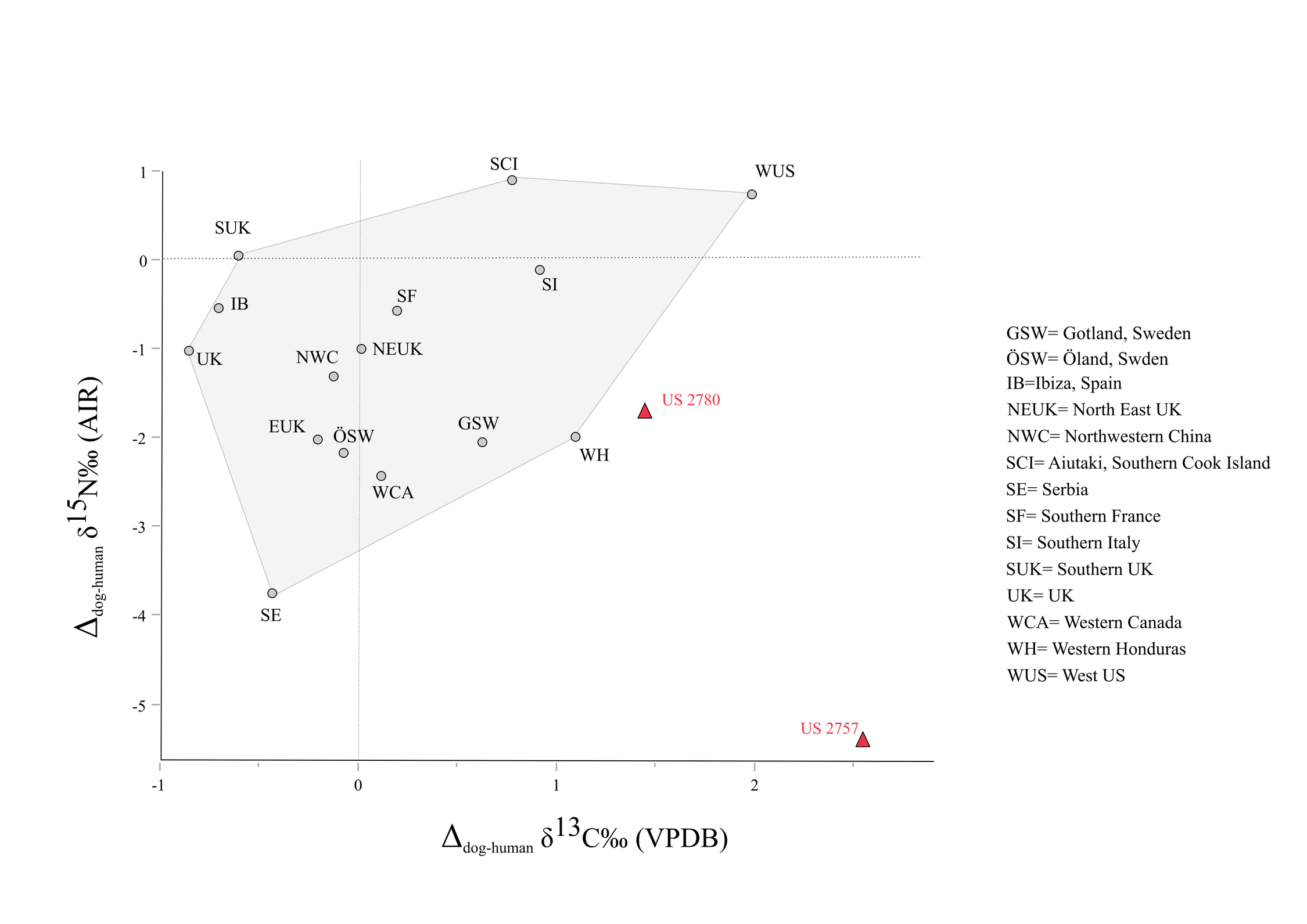

Supplement: S2 Fig — (TIF) [file pone.0293434.s010.tif]
