# Supplementary material for: "Until death do us part". A multidisciplinary study on human- Animal co- burials from the Late Iron Age necropolis of Seminario Vescovile in Verona (Northern Italy, 3rd-1st c. BCE)
Source: PLoS One. 2024 Feb 14;19(2):e0293434. doi: 10.1371/journal.pone.0293434 (PMC10866530; doi:10.1371/journal.pone.0293434)
Supplement: S1 Text — (DOCX) [file pone.0293434.s011.docx]

# **S1 Text: Methods**

## **Human ancient DNA analysis**

The inhumed individuals from *Seminario Vescovile* first underwent a molecular screening in order to assess the authenticity and preservation of the ancient DNA (aDNA). Approximately 150 mg of powdered bone was collected from the inner part of the Pars petrosa [1] (PP, isolated or still anatomically connected to the skull) for 15 human samples using a drill in a dedicated pre-PCR area of the aDNA laboratory of EURAC in Bolzano (Italy). All necessary precautions for aDNA analyses were taken during the laboratory process. DNA samples were extracted using a modified, silica-based method [2, 3] and double-stranded genomic libraries were then constructed [4] and sent to an external company (Macrogen Sequencing Centre, Seoul) for shotgun sequencing [150bp paired-end (PE) HiSeq-X systems, Illumina].

In addition, 11 available samples were analyzed for more than 1.3 million SNPs (see S4 Table) using the in-solution target capture kit "Twist Ancient DNA" (Twist Bioscience) optimized for aDNA [5]. The original protocol [6] was modified by reducing the post-enrichment amplification cycle number from 23 to 15. Then, the enriched samples were sequenced under the same conditions as the shotgun sequencing (for all laboratory work raw data, please refer to S4 Table).

The fastq files from different libraries and sequencing runs (shotgun and capture) were merged into a single fastq file for each sample. The total reads were then trimmed and merged using fastp [7]. Using BWA [8], the reads were aligned to both the Genome Reference Consortium Human Build 37 (hg19 [9]) and the revised Cambridge Reference Sequence (rCRS [10]) of the mitochondrial DNA (mtDNA) with a minimum mapping quality set at 30. Duplicate reads were removed using Dedup [11]. The MapDamage software was used to track and quantify damage patterns among the ancient reads, including fragmentation and misincorporation patterns [12] (S1 Fig). Contamination estimates from modern human DNA were inferred for all samples using mtDNA data (Schmutzi) [13] and X-chromosome data (ANGSD) [14] was additionally applied to males.

The genetic sex was estimated using both capture and shotgun data by calculating the ratio of sequences aligning to the X- and Y-chromosomes [15, 16].

Biological relatedness (kinship) among the individuals from *Seminario Vescovile* was inferred using three different methods developed especially for low-coverage aDNA data: READ, TKGWV2, and KIN. The first (READ, Relationship Estimation from Ancient DNA [17]) calculates pairwise mismatch rates in non-overlapping windows across the entire genome using pseudo-haploid data. Normalization was carried out using standard parameters. TKGWV2 [18], on the other hand, utilizes genotype likelihoods and population allele frequencies of genome-wide variants present in the 1000 Genomes Project Phase 3. These two methods allow us to infer genetic relatedness only up to the 2^nd^ degree. Lastly, KIN [19], which uses a Hidden-Markov-Model-based approach, can identify kinship up to the 3^rd^-degree and distinguishes between sibling and parent-child relationships.

## **Radiocarbon analysis**

The preparation of the bones followed Szidat et al. [20] with a slight modification that included an ultrafiltration step, as recently carried out in Steuri et al. [21]. The bones were cleaned by ultra-sonication in ultra-pure water and ground to 0.5–1 mm with a ball mill. The chemical treatment included the following steps: 0.5 mol/L hydrochloric acid (HCl) for 60 hr, 0.25 mol/L sodium hydroxide (NaOH) for 1 hr, 0.5 mol/L HCl for 1 hr, followed by a gelatinization in diluted HCl at pH 3 and 60°C overnight. The warm solution was filtered using pre-cleaned Ezee-Filters and ultrafiltration was performed with Vivaspin™ 15 30 kDa molecular weight cut-off (MWCO) ultrafilters (Sartorius).The high-molecular-weight fraction was then lyophilized and the extracted collagen was combusted and graphitized with automated graphitization equipment (AGE). The ^14^C measurements were performed with the accelerator mass spectrometry (AMS) system MICADAS using ^14^C-free sodium acetate and the NIST standard oxalic acid II (SRM 4990C) for blank subtraction, standard normalization, and correction for isotope fractionations [22].

## **Isotope ratios analyses**

The extraction of collagen for the isotopic analysis was performed at the Department of Physical Anthropology of the University of Bern from cranium and femur samples following an acid–base–acid extraction method modified after Ambrose [23, 24], DeNiro [25], and Longin [26]. The isotope ratios of carbon (^13^C/^12^C) and nitrogen (^15^N/^14^N) were measured by isotope ratio mass spectrometry at Isolab GmbH, Schweitenkirchen, Germany. An average of three measurements per sample was provided and used for subsequent analyses. Results are reported in δ-notation in units of per mill (‰) according to the international standards of Vienna Pee Dee Belemnite (V-PDB) for carbon and Ambient Inhalable Reservoir (AIR) for nitrogen. In addition, the laboratory internal standards STD R (collagen from cowhide from the EU project TRACE) and STD BRA (collagen from Brazilian cowhide) were also reported for most samples. Internal analytical errors were recorded as ± 0.1‰ for δ^13^C and ± 0.2‰ for δ^15^N (standard error of the means calculated from 3 or 4 measurements). We selected samples with a value of >1% collagen portion of dry bone (wt % = amount of extracted collagen/amount of bone powder used for extraction × 100). The molar C:N ratio ([%C/%N] × [14.007/12.011]) in the range of 2.9–3.6 was considered as good quality [25]. As good quality was considered as well, when % C was in the range of 30%–47% and % N in the range of 11%–17.3% [23, 24, 27].

**References**

1. Pinhasi R, Fernandes D, Sirak K, Novak M, Connell S, Alpaslan-Roodenberg S, et al. Optimal Ancient DNA Yields from the Inner Ear Part of the Human Petrous Bone. PLoS One. 2015;10(6):e0129102. doi: 10.1371/journal.pone.0129102.

2. Damgaard PB, Margaryan A, Schroeder H, Orlando L, Willerslev E, Allentoft ME. Improving access to endogenous DNA in ancient bones and teeth. Sci Rep. 2015;5:11184. doi: 10.1038/srep11184.

3. Rohland N, Siedel H, Hofreiter M. A rapid column-based ancient DNA extraction method for increased sample throughput. Mol Ecol Resour. 2010;10(4):677-83. doi: 10.1111/j.1755-0998.2009.02824.x.

4. Meyer M, Kircher M. Illumina sequencing library preparation for highly multiplexed target capture and sequencing. Cold Spring Harb Protoc. 2010;2010(6):pdb prot5448. doi: 10.1101/pdb.prot5448.

5. Rohland N, Mallick S, Mah M, Maier R, Patterson N, Reich D. Three assays for in-solution enrichment of ancient human DNA at more than a million SNPs. Genome Res. 2022;32(11-12):2068-78 doi: 10.1101/gr.276728.122.

6. Bioscience T. Twist Target Enrichment Standard Hybridization v1 Protocol For use with the Twist NGS Workflow.2022. Available from: https://www.twistbioscience.com/resources/protocol/twist-target-enrichment-standard-hybridization-v1-protocol.

7. Chen S, Zhou Y, Chen Y, Gu J. fastp: an ultra-fast all-in-one FASTQ preprocessor. Bioinformatics. 2018;34(17):i884-i90. doi: 10.1093/bioinformatics/bty560.

8. Li H, Durbin R. Fast and accurate long-read alignment with Burrows-Wheeler transform. Bioinformatics. 2010;26(5):589-95. doi: 10.1093/bioinformatics/btp698.

9. International Human Genome Sequencing C. Finishing the euchromatic sequence of the human genome. Nature. 2004;431(7011):931-45. doi: 10.1038/nature03001

10. Andrews RM, Kubacka I, Chinnery PF, Lightowlers RN, Turnbull DM, Howell N. Reanalysis and revision of the Cambridge reference sequence for human mitochondrial DNA. Nat Genet. 1999;23(2):147. doi: 10.1038/13779.

11. Peltzer A, Jager G, Herbig A, Seitz A, Kniep C, Krause J, et al. EAGER: efficient ancient genome reconstruction. Genome Biol. 2016;17:60. doi: 10.1186/s13059-016-0918-z.

12. Jonsson H, Ginolhac A, Schubert M, Johnson PL, Orlando L. mapDamage2.0: fast approximate Bayesian estimates of ancient DNA damage parameters. Bioinformatics. 2013;29(13):1682-4. doi: 10.1093/bioinformatics/btt193.

13. Renaud G, Slon V, Duggan AT, Kelso J. Schmutzi: estimation of contamination and endogenous mitochondrial consensus calling for ancient DNA. Genome Biol. 2015;16:224. doi: 10.1186/s13059-015-0776-0.

14. Korneliussen TS, Albrechtsen A, Nielsen R. ANGSD: Analysis of Next Generation Sequencing Data. BMC Bioinformatics. 2014;15(1):356. doi: 10.1186/s12859-014-0356-4.

15. Mittnik A, Wang CC, Svoboda J, Krause J. A Molecular Approach to the Sexing of the Triple Burial at the Upper Paleolithic Site of Dolni Vestonice. PLoS One. 2016;11(10):e0163019. doi: 10.1371/journal.pone.0163019.

16. Skoglund P, Storå J, Götherström A, Jakobsson M. Accurate sex identification of ancient human remains using DNA shotgun sequencing. J Archaeol Sci. 2013;40(12):4477-4482. doi: 10.1016/j.jas.2013.07.004.

17. Monroy Kuhn JM, Jakobsson M, Gunther T. Estimating genetic kin relationships in prehistoric populations. PLoS One. 2018;13(4):e0195491. doi: 10.1371/journal.pone.0195491.

18. Fernandes DM, Cheronet O, Gelabert P, Pinhasi R. TKGWV2: an ancient DNA relatedness pipeline for ultra-low coverage whole genome shotgun data. Sci Rep. 2021;11(1):21262. doi: 10.1038/s41598-021-00581-3.

19. Popli D, Peyregne S, Peter BM. KIN: a method to infer relatedness from low-coverage ancient DNA. Genome Biol. 2023;24(1):10. doi: 10.1186/s13059-023-02847-7.

20. Szidat S, Vogel E, Gubler R, Lösch S. Radiocarbon Dating of Bones at the LARA Laboratory in Bern, Switzerland. Radiocarbon. 2017;59(3):831-42. doi: 10.1017/rdc.2016.90.

21. Steuri N, Milella M, Martinet F, Raiteri L, Szidat S, Lösch S, et al. First Radiocarbon Dating of Neolithic Stone Cist Graves from the Aosta Valley (Italy): Insights into the Chronology and Burial Rites of the Western Alpine Region. Radiocarbon. 2023;65(2):521-538. doi: 10.1017/rdc.2023.12.

22. Szidat S, Salazar GA, Vogel E, Battaglia M, Wacker L, Synal H-A, et al. ^14^C Analysis and Sample Preparation at the New Bern Laboratory for the Analysis of Radiocarbon with AMS (LARA). Radiocarbon. 2014;56(2):561-566. doi: 10.2458/56.17457.

23. Ambrose SH. Preparation and characterization of bone and tooth collagen for isotopic analysis. J Archaeol Sci. 1990;17(4):431-451. doi: 10.1016/0305-4403(90)90007-r.

24. Ambrose SH. Isotopic analysis of paleodiets: Methodological and interpretive considerations. In: Sandford MK, editor. Investigations of ancient human tissue: Chemical analyses in anthropology Philadelphia: Gordon and Breach Science Publishers; 1993. pp. 59– 130.

25. DeNiro MJ. Postmortem preservation and alteration of in vivo bone collagen isotope ratios in relation to palaeodietary reconstruction. Nature. 1985;317(6040):806-809. doi: 10.1038/317806a0.

26. Longin R. New method of collagen extraction for radiocarbon dating. Nature. 1971;230(5291):241-242. doi: 10.1038/230241a0.

27. van Klinken GJ. Bone Collagen Quality Indicators for Palaeodietary and Radiocarbon Measurements. J Archaeol Sci. 1999;26(6):687-695. doi: 10.1006/jasc.1998.0385.
