# Supplementary material for: "Until death do us part". A multidisciplinary study on human- Animal co- burials from the Late Iron Age necropolis of Seminario Vescovile in Verona (Northern Italy, 3rd-1st c. BCE)
Source: PLoS One. 2024 Feb 14;19(2):e0293434. doi: 10.1371/journal.pone.0293434 (PMC10866530; doi:10.1371/journal.pone.0293434)
Supplement: S2 Text — (DOCX) [file pone.0293434.s012.docx]

# **S2 Text: details on burials with food offerings and additional zooarchaeological information**

## **List of burials with food offerings**

Burial 1 included a young adult (20-35 years old) male (US 3159) in a simple pit grave. The individual was placed on his left side in a flexed position and oriented north-south with his head to the north. Associated with this burial were the left hind limb bones (femur, patella, tibia, fibula, and calcaneus) of a pig (US 3159/01), whose sex was not assessable, aged ca. 3.5 years.

Burial 18 corresponded to the inhumation of a perinate (pre-term: 34-36 weeks) (US 2729) in a simple pit grave with a poorly distinguishable cut and fill. The individual was probably oriented east-west in a hyper-flexed position and the bone preservation is poor -- only skull fragments and few vertebrae and ribs are available. A large pebble, possibly serving as burial marker, was placed in the northern section of the pit. Associated with this burial were a second phalanx from an adult pig (US 2729a) and additional, unidentified faunal fragments (US 2729b).

Burial 53 included an old adult (>50 y) female (sex genetically determined) (US 2603) lying supine in a simple pit oriented north-south (head to the north, facing east) with the upper and lower limbs in extension. Two ceramic vessels placed to the right of the skull are the only grave goods. Located on a ceramic lid associated with the inhumation were the remains of the left hind limb (tibia, fibula and talus) from a ca. 3.5-year-old pig (US 2603/04) whose sex was not assessable (S4 Fig). Interestingly, this case is similar to the recently published food offering identified in the Celtic tomb of Casona di Nogara [1], another Latenian funerary context from the same region as SV. In this example, the food offering was classified as a "pork knuckle" -- a cut of meat that remains common in modern cuisine.


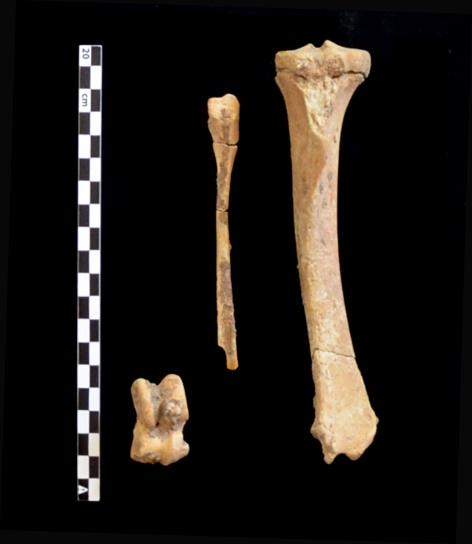


**S3 Figure. B53, US 2603/04: pig bone remains ("pork knuckle")**

Burial 84 contained an old adult (>50 y) male (US 2894) in an earthen pit with a "secco" structure consisting of aligned pebbles partially delimiting the pit. The individual was laid in a supine position oriented northeast - southwest (head to the northeast) with the upper and lower limbs in extension. Grave goods included a ceramic vessel, an iron object, and a bronze coin placed on the pelvis. Pig remains (US 2903) were located in the grave to the east of the individual's cranium. The faunal finds include the mandible of a female pig with an erupting M3 and a well-worn Pd4. This points to an age of ca. 18-24 months (Fig. S4). Additional pig remains (right hemi-mandible and a fragment of the left, right portion of maxilla, and rib fragments) were placed on the eastern side of the burial space near the cranium of individual US 2894.


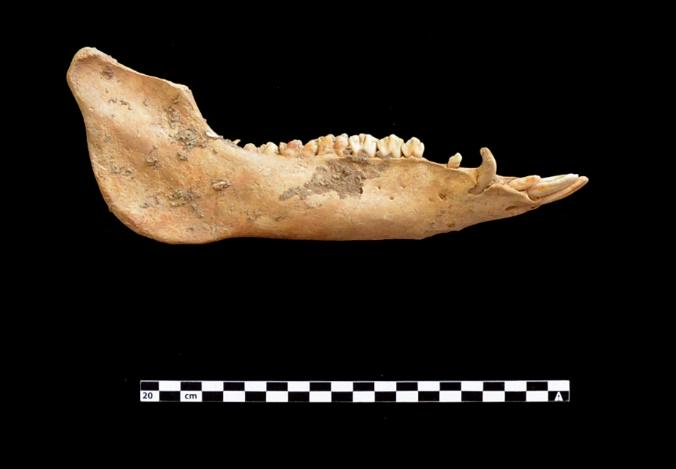


**S4 Figure. B84, US 2903: mandible of a female pig.**

Burial 97 was that of a female infant (sex determined genetically) aged between 8-16 months (US 3190) and placed supine in an earthen pit oriented northwest-southeast (head to the northwest). The skeleton is poorly preserved, partially articulated, and incomplete. The burial contained eleven grave good elements arranged to the north and west of the skull and body, including several vessels, a worked-bone item, an iron fibula placed under the sternum, and a bronze coin under the pelvis. The associated faunal remains were the rib of a pig (US 3190/05) whose age and sex were undetermined.

Burial 99 contained a young adult (20-35 years old) male (US 325) placed supine in a simple pit and oriented northwest-southeast (head to the north, facing east). The conservation of the skeleton was good, with the bones having maintained their anatomical connections. The upper and lower limbs were adducted and extended bilaterally with the scapulae rotated medially. These features suggest a lateral compression on the body, possibly due to the use of a shroud. The six recovered grave goods include ceramic vessels, a fibula, a worked bone artifact placed on the left arm, and a bronze coin under the thorax. Associated with this burial are a pelvic fragment and femur with unfused epiphyses, likely from a small, young, domestic ruminant, possibly *caprinae* (US 3251/06). The sex was not assessable.

Burial 100 lacked human remains, probably due to their removal by a later cut. The grave goods consisted of fourteen items, including ceramic vessels, metal objects, and a possible razor. The associated faunal remains are an unidentified skeletal fragment (US 3199/09).

Burial 115 included a female infant (sex determined genetically) aged between 6-9 months (US 3212) placed in simple pit, supine, and oriented north-south (head to the north, facing east). The skeleton was partially disturbed by root movement and the feet bones are almost completely missing. Six grave goods were present: two ceramic items to the northwest of the skull, two bronze fibulae located on the left arm and thorax, and a bronze coin placed under the right hand. An unidentified animal vertebra (US 3212/06) was also associated with this burial.

Burial 117 was the inhumation of an infant aged 2-3 months (US 3178) buried in a pit covered by pebbles and tuff blocks and bordered by stones, possibly the remnants of a structure. The skeleton was oriented north-south with the head to the north and possibly facing east. The burial was heavily disturbed, thus hampering a reconstruction of the infant's original position. A rich set of grave goods included four ceramic vessels and two bronze coins. Associated with this burial were parts of the left hind limb of an adult pig whose sex was not assessable (US 3178a) (2^nd^ and 3^rd^ metatarsals, cuboid, 2^nd^ accessory phalanx), plus an additional four unidentified fragments probably related to the same pig.

Burial 131 is the inhumation of a middle adult (36-50 years old) female (US 3267), placed supine and with the torso slightly rotated to the right and oriented northeast-southwest (head toward the northeast, facing east) in a pit lined by river pebbles on its southern side. These pebbles are likely the remnants of a funerary structure. The bones were well preserved and the skeleton maintained the original anatomical connections. The burial included four grave goods, among which were a globular jar laid presumably upside down and placed to the north of the skull and a bronze coin under the skull. The associated faunal remains is an undetermined pig bone (US 3267/03).

Burial 147 is that of an old adult (>50 years old) woman (US 3989) placed supine and extended in a simple pit. The pit was lined along its perimeter by a row of stones (US 3988) and featured a covering of stones and pebbles (US 3982) which were probably originally placed on a wooden plank covering the tomb. The body was oriented north-south with the head to the north. Grave goods included ceramic vessels, 2 coins, and an iron fibula. Associated with this burial was the near-complete skeleton of a chicken (*Gallus gallus domesticus*) (US 3989/09), missing only the beak and the distal part of the feet. In proximity to the bird were two terrestrial snail shells (*Pomatias elegans* and *Cepaea nemoralis*) (US 3989a-b), though the presence of these two items could be due to a later intrusion.

Burial 148 contained the remains of a young adult (20-35 years old) male (US 3231) placed supine in a simple pit and oriented north-south (head to the north, facing east). Only the skull of the individual was recovered upon excavation; the postcranial skeleton exceeded the southern limit of the excavation area and was therefore unable to be retrieved. Eleven grave goods were arranged in a semicircular pattern immediately to the north and east of the skull. These objects included mainly ceramic vessels as well as the blade of an iron knife partially in contact with the cranium. The associated faunal find is a pig rib (US 3231/10).

## **Additional zooarchaelogical information**

S8 Tables report the osteometric measurements (mm) of the faunal remains (after [2]) subdivided by species. S7 Table presents the unpublished measurements of the animal remains from grave 7 of Lazisetta mentioned in the main text [3].

The following is a list of additional animal bones that, even if present, could not be measured due to their poor preservation.

### **Addendum: Lazisetta di Santa Maria di Zevio, grave 7 (T7)** (S7 Table)

Tb 7/90:

Right fibula (fragmentary)

Centrotarsal

### **Pig** (S8c Table)

US 3178a

2nd left metatarsal

Left cuboid

2^nd^ supernumerary phalanx

4 unidentified bone fragments – possibly of *Sus domesticus*

### **Chicken** (Table S8e)

US 3989/09

1 f di forcella

4 sternal fragments

2 pelvic fragments

3 vertebrae

2 rachis fragments

13 rib fragments

1 ulnar fragment

2^nd^ digital phalanx

**Horse**

Burial 102 (see main text) also revealed in its fill a horse metatarsal whose length suggests a withers height of 147 cm. The presence of this bone in the burial fill may have originated from a post-depositional disturbance that occurred during later (Roman) times. Accordingly, this specimen was not included in our analyses and is not reported in Table 1.

**References**

1. Salzani L, Cavazzuti C, Tecchiati U. Tomba celtica della fase di romanizzazione dalla località Casona di Nogara (Verona). In: Cresci G, Gambacurta G, editors. Il dono di Altino Scritti di archeologia in onore di Margherita Tirelli. Venezia: Università degli studi di Venezia, Ca’ Foscari; 2019. pp. 11-24.

2. von den Driesch A. A guide to the measurement of animal bones from archaeological sites: as developed by the Institut für Paläoanatomie, Domestikationsforschung und Geschichte der Tiermedizin of the University of Munich. Harvard: Harvard University Press; 1976.

3. Salzani L. Una tomba a carro. In: Aspes A, editor. Preistoria veronese contributi e aggiornamenti. 5. Verona: Memorie museo civico di storia naturale di Verona, sezione scienze dell’uomo; 2002. pp. 203-204.
